# Bimodal activity of diurnal flower visitation at high elevation

**DOI:** 10.1002/ece3.8074

**Published:** 2021-09-02

**Authors:** Xin Xu, Zong‐Xin Ren, Judith Trunschke, Jonas Kuppler, Yan‐Hui Zhao, Eva Knop, Hong Wang

**Affiliations:** ^1^ Key Laboratory for Plant Diversity and Biogeography of East Asia Kunming Institute of Botany Chinese Academy of Sciences Kunming China; ^2^ University of Chinese Academy of Sciences Beijing China; ^3^ Yunnan Lijiang Forest Ecosystem National Observation and Research Station Lijiang China; ^4^ Institute of Evolutionary Ecology and Conservation Genomics Ulm University Ulm Germany; ^5^ Agroecology and Environment Agroscope Zürich Switzerland; ^6^ Department of Evolutionary Biology and Environmental Studies University of Zürich Zürich Switzerland

**Keywords:** *Bombus*, diurnal activity patterns, flower visitors, high elevation, solar radiation

## Abstract

Successful pollination in animal‐pollinated plants depends on the temporal overlap between flower presentation and pollinator foraging activity. Variation in the temporal dimension of plant–pollinator networks has been investigated intensely across flowering seasons. However, over the course of a day, the dynamics of plant–pollinator interactions may vary strongly due environmental fluctuations. It is usually assumed there is a unimodal, diurnal, activity pattern, while alternative multimodal types of activity patterns are often neglected and deserve greater investigation. Here, we quantified the daily activity pattern of flower visitors in two different habitats contrasting high elevation meadows versus forests in Southwest China to investigate the role of abiotic conditions in the temporal dynamics of plant–pollinator interactions. We examined diurnal activity patterns for the entire pollinator community. Pollinator groups may differ in their ability to adapt to habitats and abiotic conditions, which might be displayed in their patterns of activity. We hypothesized that (a) pollinator communities show multimodal activity patterns, (b) patterns differ between pollinator groups and habitat types, and (c) abiotic conditions explain observed activity patterns. In total, we collected 4,988 flower visitors belonging to six functional groups. There was a bimodal activity pattern when looking at the entire pollinator community and in five out of six flower visitor groups (exempting solitary bees) regardless of habitat types. Bumblebees, honeybees, dipterans, lepidopterans, and other insects showed activity peaks in the morning and afternoon, whereas solitary bees were most active at midday. Activity of all six pollinator groups increased as solar radiation increased and then decreased after reaching a certain threshold. Our findings suggest that in habitats at higher elevations, a bimodal activity pattern of flower visitation is commonly employed across most pollinator groups that are diurnal foragers. This pattern may be caused by insects avoiding overheating due to elevated temperatures when exposed to high solar radiation at midday.

## Graphical abstract

We found a general bimodal activity of diurnal flower visitors under extreme conditions at high elevation. This bimodal pattern positively relates with solar radiation. These findings suggest that in high elevation environments, a bimodal activity pattern of flower visitation is commonly employed across flower visitor groups and may be caused by insects avoiding overheating due to elevated temperatures when exposed to high solar radiation during midday.

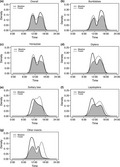

## 1 INTRODUCTION

Interactions between plants and their pollinators determine the assemblage and function of communities at various temporal and spatial scales (Artz et al., 2010; Barônio & Torezan‐Silingardi, 2017; Price et al., 2005; Venjakob et al., 2016). Interactions between flowers and their pollinators are shaped by varying degrees of coevolution/coadaptation resulting in matches of floral and floral forager traits (Faegri & van der Pijl, 2013; Fenster et al., 2004; Leal et al., 2020; Proctor et al., 1996; Zych et al., 2019). For coadaptation to occur, the temporal and spatial dimensions of plant–pollinator interactions must be self‐consistent (Trøjelsgaard & Olesen, 2016; Valverde et al., 2016).

However, the temporal dimensions defining plant–pollinator interactions can vary from short to long periods (CaraDonna et al., 2021; Schwarz et al., 2020). In a community, both plants and associated pollinators display a seasonal pattern of flowering, versus adult emergence and foraging cycles, respectively. It is expected this causes temporal variations and seasonal turnovers in the pollinator community and plant–pollinator networks (Cane et al., 2005; Herrera, 1988; Pyke et al., 2011; Biella et al., 2017; Souza et al., 2018; Rabeling et al., 2019).

Some plant species follow a diurnal rhythm of flowering and that can have a strong influence on plant–pollinator interactions (Fründ et al., 2011), contributing to distinct patterns of foraging activity among different pollinator groups (Knop, Gerpe, et al., 2017; Zoller et al., 2020).

In contrast, plant–pollinator interactions may also be driven by the circadian rhythms of pollinators as determined by comparative physiologies constrained by phylogenetic relationships (Beer & Bloch, 2020; Bloch, 2010; Bloch et al., 2017; Heinrich, 1979). Most pollinators are diurnal such as bees, butterflies, flies, and birds, but some moths, beetles, and bats forage and pollinate along crepuscular‐nocturnal rhythms (Knop, Gerpe, et al., 2017; Knop, Zoller, et al., 2017; Willmer, 2011). The foraging of bumblebees and honeybees typically follows strong circadian rhythms. Most bee species rely on the circadian clocks to predict sunrise and sunset, as they adopt higher levels of activity during the day (Chittka et al., 2013; Vaudo et al., 2014; Yerushalmi et al., 2006) to make the most efficient use of sufficient sunlight to find preferred flowers for foraging (Bloch et al., 2017).

To our knowledge, most previous research investigating patterns of foraging activities of flower visitors has focused either on a single‐few focal plant species or a subset of insect taxa (Gottlieb et al., 2005; Sgolastra et al., 2016; Souza et al., 2017). Past studies of the temporal dynamics of plant–pollinator interactions focused primarily on seasonal and year‐to‐year variability (Chacoff et al., 2018; Dupont et al., 2009). Therefore, the temporal dimensions of plant–pollinator interactions at a community level remain less well studied, particularly at finer resolutions during the course of a floral day (but see Baldock et al., 2011; Knop, Gerpe, et al., 2017; Schwarz et al., 2021; Zoller et al., 2020).

While the dynamics of plant–pollinator interactions are dependent on internal rhythms (Bloch et al., 2017; Fenske et al., 2018; see above), there are additional and extrinsic parameters, such as the availability of plant resources and/or environmental conditions to determine plant‐pollinator interaction dynamics (Schwarz et al., 2021). Among these extrinsic parameters, abiotic parameters include ambient temperature, solar radiation, relative humidity, and wind speed are the best‐studied (Sanderson et al., 2015; Sgolastra et al., 2016; Willmer, 2011). Indeed, visitors adapt their foraging activity to optimize the time of resource collection during the day to maximize their efficiency according to the quality of environmental conditions (Cook et al., 2011; Schäffler & Dötterl, 2011; Stone et al., 1999). Different groups of pollinators may show different adaptations to the same environmental factors (Knop, Gerpe, et al., 2017; Sgolastra et al., 2016; Tuell & Isaacs, 2010; Vicens & Bosch, 2000). For example, in a study of the pollination of *Vaccinium corymbosum*, honeybees (*Apis mellifera*) were most active under conditions of warm temperatures and high solar radiation provided wind velocity and relative humidity were low. In contrast, bumblebees (*Bombus impatiens*) were the dominant pollinators during periods of poor weather (Tuell & Isaacs, 2010).

Furthermore, it is usually assumed that there is a unimodal pattern of pollinator activities (e.g., Baldock et al., 2011; Knop, Gerpe, et al., 2017) but Zoller et al. (2020) suggested recently that flower visitors might switch from unimodal to a bimodal within the Arctic Circle. It is thus possible, with more field studies, bimodal or even multimodal patterns of activity in pollination communities may be more common than it is previously anticipated.

As extrinsic abiotic parameters usually vary between or even within habitats, the general habitat context may be a key factor governing spatio‐temporal variation in flower visitation patterns (Jha & Vandermeer, 2009). A recent study showed that warmer microhabitats positively affected the richness of flower visitors in alpine communities in Austria (Ohler et al., 2020). Therefore, the pollinator‐specific differences in flower visitation patterns might vary depending on habitat type.

Heterogenous subalpine and alpine environments in the Himalaya‐Hengduan Mountains occur at extremely high elevations (e.g., 1,300–6,000 m) along a complex mountain topology, while harboring unusually high plant and insect diversities (Ren et al., 2018; Xing & Ree, 2017). Previous studies in this region revealed that eusocial bumblebees and the native honeybee (*Apis cerana*) dominate flower communities that are characterized by generalized pollination systems (Liang et al., 2018; Zhao et al., 2019).

In such mountain environments, atmospheric temperature decreases while solar radiation increases as elevation increases (Körner, 2007). Therefore, previous studies focused specifically on the effect of temperature and solar radiation (Birrell et al., 2020; Hodkinson, 2005). They found that increasing temperature resulted in increasing flight departures of honeybees up to a certain threshold of solar radiation after which there was a negative response of flight activity to temperature (Burrill & Dietz, 1981). Furthermore, temperature effects can differ between different insect species or their different morphologies (Cena & Clark, 1972). We should expect, therefore, that at high elevations with high degrees of solar radiation both factors must shape the daily patterns of pollinator activity.

As Himalayan mountains are characterized by extreme weather conditions while harboring diverse habitats, they provide ideal settings to test whether diurnal pollinator activities in communities show bi‐ or multimodal activity patterns under high solar radiation. To obtain a more generalized understanding, our investigation was conducted in two contrasting habitats. We hypothesized that, under conditions of extreme high solar radiation at high elevations, (a) the visitation patterns are bi‐ or multimodal but differ for different groups of flower visitors and habitat types, and (b) abiotic factors explain the activities of each pollinator group when combined or addressed independently. We assessed variation in the diurnal activity patterns of different visitor groups across meadow (exposed) and forest (shaded) transects at an elevation belt of 3,200 m on the Yulong Snow Mountain, SW China. We asked the following four questions: (a) Do flower visitors in high elevation environments show a bimodal or multimodal visitation pattern during the course of the day? (b) Does the pattern differ between flower visitor groups? (c) Which abiotic factors, that are, relative humidity, wind velocity, and solar radiation, influence variation in the relative abundance of visitors during the day? (d) Do flower visitation patterns vary between meadow and forest habitats?

## 2 MATERIALS AND METHODS

### 2.1 Study location and system

This study was conducted at Yulong Snow Mountain near the city of Lijiang, Northwestern Yunnan, China (27°00′N, 100°10′E), for two consecutive flowering seasons (2018 and 2019). The study area is located within the Himalaya‐Hengduan Mountains region, which differs from other mountain ranges worldwide because of its higher alpine tree line from 3,900 m to 4,900 m (Lu et al., 2021). The region is characterized by a warm, rainy season from May to October and a colder dry season, with periodic snow storms, from November to April. Our study sites are located in the Yunnan Lijiang Forest Ecosystem National Observation and Research Station at an elevation around 3,200 m. It is a mosaic of wet meadows and drier pine‐oak forests. During the two study periods from July to August, the average temperature was 13.0 and 13.3°C. Average relative humidity was 91.8 and 89.7%. The average solar radiation was 89.5 and 95.5 μmol·m^−2^·s^−1^, with an average precipitation of 11.2 and 14.0 mm in 2018 and 2019, respectively.

### 2.2 Sampling of plant–insect interactions

We chose three sites with more than 1.0 km distance between any two locations. In each site, we set up two different habitat transects, one in the meadow and a parallel transect in the adjacent forest. Each transect was 100 m in length and 3 m wide. The distance between the two habitat transects exceeded 50 m. In total, six transects were sampled and repeated for 2 years (Table S1). The floral community composition was relatively similar between habitats (Xu et al., unpublished data).

In both study seasons, we observed plant and visitor interactions during daylight hours. Sampling was performed on a total of 14 sunny days (8 days in 2018, 6 days in 2019) excluding days of strong wind, fog, or rainfall. On all sampling days, we collected data in all six transects simultaneously between 08:00 and 20:00 with one person sampling per transect. When an insect arrived at a flower and contacted the plant's reproductive structures, while actively foraging for pollen and/or nectar, we recorded this as one interaction. This included recording each insect observation the date, time, and plant species on which it foraged. Each flower visitor recorded was then collected, stored in a separate 2‐ml Eppendorf tube containing 95% ethanol. The specimen was taken to the laboratory for identification. Following identification, specimens were pinned and vouchers deposited at the Kunming Institute of Botany, Chinese Academy of Sciences.

As the dominant foragers were bumblebees (*Bombus* spp.) and honeybees (*A. cerana*), they were identified as species (like the bumblebees in Yulong Snow Mountain, *Bombus friseanus* and *Bombus lepidus*). Remaining taxa were identified to genus (e.g., *Andrena*, *Musca*) or Order (Coleoptera). Insect specimens were segregated into “functional groups,” which are defined here as those visitors that interact with the same flowers in a similar manner. We used criteria of the same Order, genus and species, similarity in body size and foraging behavior to identify six functional visitor groups in our study. They are defined here as bumblebee, honeybee, solitary bee (i.e., bees that were not in the genera *Bombus* or *Apis* and with a body size less than 8 mm), dipteran, lepidopteran, and other insects (Hemiptera, Coleoptera).

### 2.3 Environmental abiotic factors

To investigate whether abiotic parameters affect plants and their visitor interactions, we recorded hourly averages of ambient temperature (°C), relative humidity (%), wind velocity (m/s), and solar radiation (μmol·m^−2^·s^−1^) on all sampling days from the local weather station in the meadow‐forest transition at one of our field sites, managed by Yunnan Lijiang Forest Ecosystem National Observation and Research Station. We expected that these data were representative across our study sites as all sites were approximately at 3,200 m, were located within approximately 1 km of each other, and were of the same vegetation type (i.e., meadows or forests).

### 2.4 Statistical analysis

The data were analyzed mainly by a nonparametric kernel density estimation method (Ridout & Linkie, 2009). It was assumed that insect behavior was distributed within a continuous 24‐hr time cycle, and behavior events were based on continuous random sampling. Our sampling was continuous from 8:00 to 20:00 giving us 12 hr each day to record the diurnal pattern of flower visitor abundance. To test whether the activity pattern varied significantly at different times of the day, we applied kernel density estimation using the overlap package (version 0.3.3; Ridout & Linkie, 2009). The kernel density estimation method does not require any assumptions for the data distribution. It was used to characterize the data distribution from the data set itself (Ridout & Linkie, 2009). First, the time of the day was converted to decimal format (0–1) and then converted to radian format (Ridout & Linkie, 2009). Second, we used the *densityPlot* function to draw fitted circular kernel distribution curves for each individual group of flower visitor within each habitat type. We then used the *overlapPlot* function to draw fitted circular kernel distribution overlap curves for different groups of flower visitor within the two habitat types. All kernel plots are based on the pooled data across all observation days.

We then performed an activity patterns overlap analysis to test (a) whether the diurnal activity pattern of all visitor groups together and the different flower visitor groups, respectively, consistent between the two different habitat types, and (b) whether the diurnal activity pattern of the different flower visitor groups varies within the same habitat type. The overlap coefficient was obtained by comparing each time point of the density function with a minimum of two cycle lengths (Ridout & Linkie, 2009). According to the smaller number of samples of the paired flower visitor groups, we used the *overlapEst* function of the overlap package to calculate the overlap coefficient and determine the degree of overlap in visitation patterns between the two habitat types and among the six flower visitor groups, respectively. The overlap coefficient ranges from 0 to 1 indicating a weak to strong overlap. This statistic of the overlapping degree of activity pattern, however, is purely descriptive and does not provide a threshold to verify whether the difference in activity pattern of flower visitor groups is significantly different between habitat types and different flower visitor groups in the same habitat type. To test these two predictions explicitly, we used the *compareCkern* function of the activity package (version 1.3; Rowcliffe, 2019) in order to perform a nonparametric bootstrapping (1,000 iterations) comparing activity patterns.

To determine whether any of the four abiotic factors (ambient temperature, relative humidity, solar radiation, and wind velocity) affected the abundance of flower‐visiting insects, we used generalized linear mixed models (GLMMs) implemented in the *glmmTMB* package (version 1.0.2.1; Bates et al., 2015; Brooks et al., 2017) with negative binomial error distribution and correction for zero‐inflation (i.e., the distribution does not predict as much zero as in the data) required to meet model assumptions. We ran a total of seven models, with the abundance of flower visitors and six functional visitor groups as response variables. Each model included habitat type, ambient temperature, ambient temperature^2^, relative humidity, relative humidity^2^, wind velocity, solar radiation, and solar radiation^2^ as explanatory fixed factors. Study site and sampling day were included as random factors in each model. We included the quadratic terms of temperature, relative humidity, and solar radiation in the model because high and low values in those variables might have a negative impact on flower visitor activity. The significance of fixed factors was assessed with Type III Wald chi‐square tests implemented in the *ANOVA* function of the car package (version 3.0‐10; Fox & Weisberg, 2019). To check if all model assumptions were met, we used the DHARMa package (version 0.3.3.0; Hartig, 2020) and the *check_collinearity* function of the performance package (version 0.5.0; Lüdecke et al., 2020). In addition, we ran a Pearson correlation among all the abiotic variables across the 2 years, using *corr.test* function of psych package (version 2.0.7; Revelle, 2020) to test their correlations. After running initial full GLMM models including all four variables, we removed ambient temperature due to high collinearity between temperature and solar radiation (VIF > 5). In fact, solar radiation was highest around midday (Figure S1d) and correlated strongly with temperature (*r* = 0.716, *p* < .001; Figure S2). We removed temperature rather than solar radiation even though temperatures at midday were highest (when solar radiation was also at its highest), as they had not reached temperature levels (15.1 ± 1.5°C) known to limit insect species activity (Kühsel & Blüthgen, 2015). Furthermore, due to previous research on the important role of solar radiation on honey bee activity at higher elevations (see Burrill & Dietz, 1981), we decided to retain solar radiation. Our selection was supported by the fact that models which included temperature instead of solar radiation showed a positive effect only in honeybees; therefore, it showed largely no effect on other insect activity (Table S5). Thus, the final analysis only included relative humidity, solar radiation, and wind velocity as explanatory factors.

Based on the daily bimodal pattern of flower visitor activities, we conducted separate analyses of the abiotic factors associated with each abundance peak with a random factor plotted showing the before noon (before 12:00) and after noon (after 12:00) curves except for the solitary bee group (unimodal pattern within a day). We used generalized linear mixed models (GLMMs) implemented in the *glmmTMB* package (version 1.0.2.1; Bates et al., 2015; Brooks et al., 2017) with a negative binomial error distribution. We included the quadratic terms of temperature, relative humidity, and solar radiation in the model because high and low values in those variables might have a negative impact on flower visitor activity. For each model, we also calculated the R^2^, the coefficient of determination, to see which variable explained the most variance (Nakagawa & Schielzeth, 2013). After we ran this model, the environmental variables' significance was corrected for multiple testing and calculated as *p*‐adjusted (Benjamini & Hochberg, 1995; Benjamini & Yekutieli, 2001).

All statistical analyses were calculated with the R Statistical Software (version 4.0.2; R Core Team, 2020) with the R Studio interface (version 1.3.1093; RStudio Team, 2020).

## 3 RESULTS

We documented 4,988 insect visits to 75 plant species noting that 78.3% of the insect specimens were identified to species level, while the remaining 23.5% were identified to the level of Order (Table 1). The bumblebee was most abundant making up 47.2% of all recorded flower visitations.

**TABLE 1:** The total number of flower‐visiting insects belonging to six functional groups collected in high elevation communities (meadow and forest) during two flowering seasons on Yulong Snow Mountain, SW China

Functional groups	Meadow	Forest	Sum across habitats
Bumblebee	1,878 (49.0%)	478 (41.5%)	2,356 (47.2%)
Honeybee	1,065 (27.8%)	395 (34.3%)	1,460 (29.3%)
Diptera	615 (16.0%)	147 (12.8%)	762 (15.3%)
Solitary bee	103 (2.7%)	63 (5.5%)	166 (3.3%)
Lepidoptera	86 (2.2%)	54 (4.7%)	140 (2.8%)
Other insects	89 (2.3%)	15 (1.3%)	104 (2.1%)
Total	3,836	1,152	4,988

As expected, abiotic conditions varied during the course of a day, but the patterns were similar in the two study seasons. In general, the ambient temperature was lowest early in the day, and the warmest period occurred between 2:00 and 4:00 p.m. (Figure S1a). Relative humidity peaked in the early morning and then decreased reaching its lowest values around 4:00 p.m. (Figure S1b). Wind velocity was distributed bimodally peaking at 12:00–1:00 p.m. and again at 3:00–4:00 p.m. (Figure S1c). Solar radiation showed a unimodal pattern. The strongest period of radiation occurred between 12:00 and 4:00 p.m. (Figure S1d). The results of the Pearson correlation between the later three abiotic variables showed that relative humidity was correlated negatively with wind velocity (*r* = −0.536, *p* < .001) and solar radiation (*r* = −0.641, *p* < .001). Wind velocity correlated positively with solar radiation (*r* = 0.639, *p* < .001; Figure S2).

### 3.1 Diurnal activity pattern and variation among flower visitor groups

**FIGURE 1:**
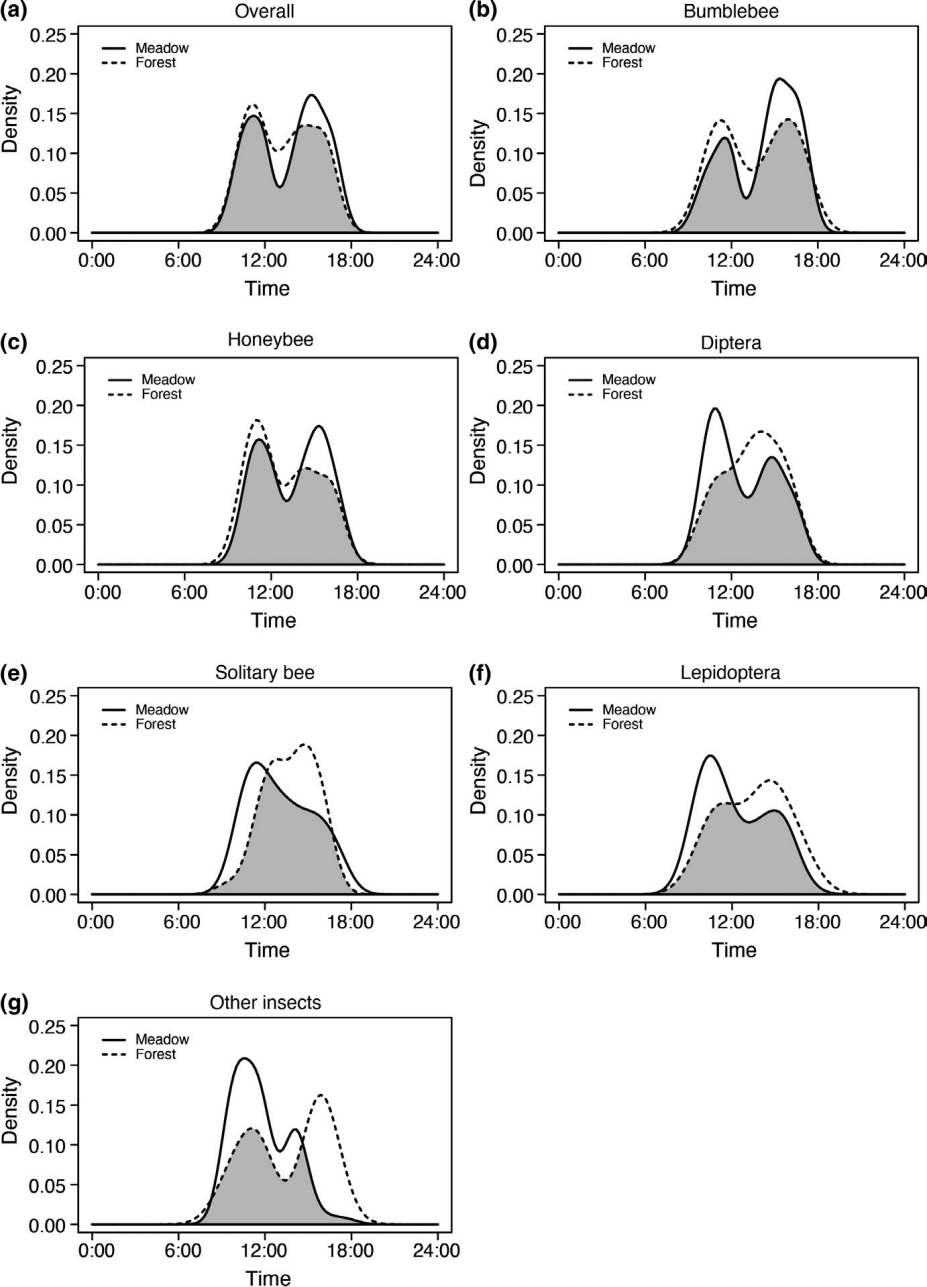
Comparison of diurnal activity patterns in overall flower visitors (a), bumblebee (b), honeybee (c), Dipteran (d), solitary bee (e), Lepidopteran (f), and other insects (g) between meadows and forests across two flowering seasons on Yulong Snow Mountain, SW China. The black curves are fitted circular kernel distributions in the meadow. Black dash curves are fitted circular kernel distributions in the forest. The overlapping coefficient equals the area in gray

We found a consistent diurnal pattern of flower visitor activity for most groups except for solitary bees. In general, visitation activities reached two peaks. The major peak occurred around 11:00 a.m., and a secondary but smaller peak appeared between 3:00 and 4:00 p.m. There was a marked decrease in activity around midday (Figure 1a; Figures S3a and S4a). In contrast, the activity of solitary bees was unimodal in both meadow and forest with noticeably different peak periods. For these insects, we detected a peak at 11:00 a.m. in the meadows and at 3:00 p.m. in the forests (Figure 1e; Figures S3e and S4e).

In the meadows, the remaining five flower visitor groups showed a similar bimodal activity pattern, but the exact timing of their activity peaks differed slightly among the groups (Figure 1; Figure S3). The overlap coefficient of activity patterns across flower‐visiting community ranged between 0.578 and 0.904 indicating medium to strong similarity among groups. Specifically, bumblebees showed a significantly different diurnal activity pattern compared to the five remaining groups (*p* < .001; Table S2). In forests, four flower visitor groups (excluding solitary bees and dipterans) showed a similar bimodal activity pattern. The exact timing of their activity peaks varied only slightly among these groups (Figure 1; Figure S4). The overlap in activity patterns varied between 0.738 and 0.941 across the different flower visitor groups indicating strong activity overlaps. Again, bumblebees had a significantly different diurnal pattern of activity compared to honeybees, dipterans, and solitary bees (*p* < .05; Table S2).

### 3.2 Effect of four abiotic factors on the abundance of flower visitor

**FIGURE 2:**
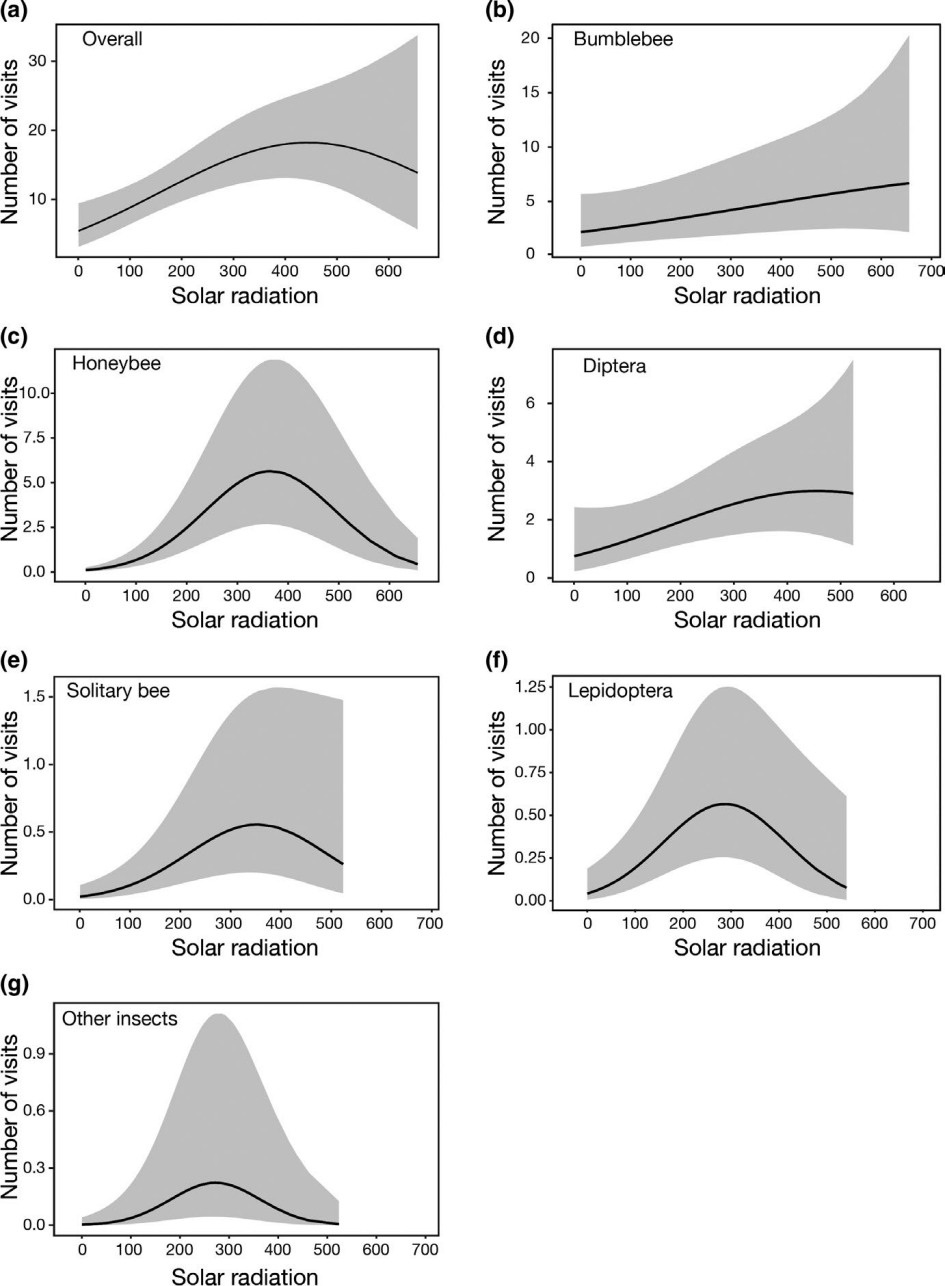
The relationship between the number of visits and solar radiation plotted from the results of the GLMMs model. (a) Overall flower visitors, (b) bumblebee, (c) honeybee, (d) Diptera, (e) solitary bee, (f) Lepidoptera, and (g) other insects. The light gray area represents the 95% confidence interval

Generally, all members of the flower visitor community together (overall flower visitors) and each separate, flower visitor group showed a significant relationship with solar radiation (*p* < .05; Table 2; Tables S3–S4). In addition, the same flower visitor groups also showed a significant negative relationship with relative humidity (*p* < .05; Table 2; Table S3). The overall abundance of flower visitors showed a unimodal concave pattern with solar radiation and relative humidity (*p* = .001 for quadratic term of solar radiation). With increasing solar radiation, the number of visitations increased until it peaked at approximately 450 μmol·m^−2^·s^−1^ and then declined (Figure 2). With increasing relative humidity, the number of visitations increased until it peaked at about 80% and then declined (Figure S6). However, wind velocity did not significantly affect the abundance of flower visitors (*p* > .05 for all terms of wind velocity; Table 2; Table S3).

**TABLE 2:** Results of the effect of environmental variables on the abundance of flower visitors in two flowering seasons on Yulong Snow Mountain, SW China

Parameter	Chi‐sq.	Df	*p* > Chi‐sq.
Overall			
Intercept	54.231	1	**<.001**
Relative humidity	2.509	1	.113
Relative humidity^2^	11.555	1	**.001**
Wind velocity	1.876	1	.171
Solar radiation	13.665	1	**<.001**
Solar radiation^2^	3.851	1	**.050**
Habitat type	53.232	1	**<.001**
Bumblebee			
Intercept	0.950	1	.330
Relative humidity	10.335	1	**.001**
Relative humidity^2^	3.928	1	**.047**
Wind velocity	0.456	1	.500
Solar radiation	4.269	1	**.039**
Solar radiation^2^	0.169	1	.681
Habitat type	10.826	1	**.001**
Honeybee			
Intercept	1.210	1	.271
Relative humidity	0.347	1	.556
Relative humidity^2^	0.725	1	.395
Wind velocity	3.572	1	.059
Solar radiation	101.843	1	**<.001**
Solar radiation^2^	53.492	1	**<.001**
Habitat type	4.486	1	**.034**
Diptera			
Intercept	1.251	1	.263
Relative humidity	1.687	1	.194
Relative humidity^2^	0.381	1	.537
Wind velocity	0.213	1	.644
Solar radiation	4.455	1	**.035**
Solar radiation^2^	1.058	1	.304
Habitat type	8.052	1	**.005**
Solitary bee			
Intercept	9.872	1	**.002**
Relative humidity	2.232	1	.135
Relative humidity^2^	0.452	1	.501
Wind velocity	0.193	1	.661
Solar radiation	18.192	1	**<.001**
Solar radiation^2^	7.071	1	**.008**
Habitat type	0.061	1	.805
Lepidoptera			
Intercept	6.185	1	**.013**
Relative humidity	1.047	1	.306
Relative humidity^2^	0.142	1	.707
Wind velocity	0.070	1	.791
Solar radiation	9.538	1	**.002**
Solar radiation^2^	8.584	1	**.003**
Habitat type	0.613	1	.434
Other insects			
Intercept	6.219	1	**.013**
Relative humidity	0.735	1	.391
Relative humidity^2^	1.531	1	.216
Wind velocity	0.005	1	.945
Solar radiation	7.986	1	**.005**
Solar radiation^2^	11.018	1	**<.001**
Habitat type	1.871	1	.171

Note

Values are derived from generalized linear mixed models, and significance was assessed with Type III Wald chi‐square tests. Significant effects at *p* < .05 are presented in bold.

Different flower visitor groups showed different responses to environmental factors. Bumblebee abundance showed a unimodal concave pattern with relative humidity (*p* < .05). The number of bumblebee visitations increased with increasing relative humidity until it peaked at about 70% and then declined again (Figure S6). However, the quadratic term of solar radiation for bumblebees (*p* = .681) and dipterans (*p* = .304) was not significant, indicating that there was no peak value but a linear increase (Figure 2b,d). In contrast, honeybee, solitary bee, lepidopteran, and other insects' abundances showed a unimodal concave pattern with solar radiation. With increasing solar radiation, the number of visitations increased until it peaked, respectively, at about 370, 350, 280, and 280 μmol·m^−2^·s^−1^ (Figure 2).

Before noon, all flower visitors including bumblebees and honeybees showed a significantly positive relationship with ambient temperature and solar radiation, but there was a negative relationship with relative humidity (Table 3; Table S6). The abundance of overall visitors showed a bell‐shaped curve with ambient temperature, relative humidity, and solar radiation (Figures S7–S10). Dipterans showed a significantly positive relationship with solar radiation (Table 3; Table S6), with their abundance showing a linear relationship with solar radiation (Figures S7‐S10).

After noon, overall flower visitors showed a significantly positive relationship with ambient temperature and solar radiation, but with a negative relationship with relative humidity (Table 3; Table S6). However, dipterans showed a significantly positive relationship with solar radiation and a negative relationship with relative humidity. Dipterans showed no relationship with ambient temperature (Table 3; Table S6). The abundance of overall visitors showed a bell‐shaped curve with relative humidity and a linear relationship with ambient temperature. The abundance of bumblebees showed a bell‐shaped curve with ambient temperature and a linear relationship with relative humidity. The abundance of honeybee showed bell‐shaped with relative humidity and linear relationship with ambient temperature. The abundance of dipterans showed a linear relationship with relative humidity (Figures S7–S10).

**TABLE 3:** Results of the effect of separating the environmental variables associated with each abundance peak independently

Functional group	Environmental variable	*p*‐adjust		Marginal R^2^	Time of the day
		Variable	Variable^2^		
Overall	Ambient temperature	**<0.001**	**<0.001**	0.137	Before noon
Overall	Ambient temperature	**<0.001**	0.062	0.196	After noon
Overall	Relative humidity	**<0.001**	**<0.001**	0.098	Before noon
Overall	Relative humidity	**<0.001**	**0.019**	0.156	After noon
Overall	Wind velocity	**<0.001**	—	0.045	Before noon
Overall	Wind velocity	**<0.001**	—	0.116	After noon
Overall	Solar radiation	**<0.001**	**<0.001**	0.126	Before noon
Overall	Solar radiation	**<0.001**	**<0.001**	0.372	After noon
Bumblebee	Ambient temperature	**<0.001**	**0.007**	0.135	Before noon
Bumblebee	Ambient temperature	**<0.001**	**0.011**	0.231	After noon
Bumblebee	Relative humidity	**<0.001**	**0.003**	0.103	Before noon
Bumblebee	Relative humidity	**<0.001**	0.073	0.161	After noon
Bumblebee	Wind velocity	**<0.001**	—	0.058	Before noon
Bumblebee	Wind velocity	**<0.001**	—	0.09	After noon
Bumblebee	Solar radiation	**<0.001**	**0.006**	0.119	Before noon
Bumblebee	Solar radiation	**<0.001**	**<0.001**	0.284	After noon
Honeybee	Ambient temperature	0.099	**0.009**	0.069	Before noon
Honeybee	Ambient temperature	**<0.001**	0.163	0.123	After noon
Honeybee	Relative humidity	0.153	**0.025**	0.043	Before noon
Honeybee	Relative humidity	**<0.001**	**0.042**	0.088	After noon
Honeybee	Wind velocity	**0.032**	—	0.019	Before noon
Honeybee	Wind velocity	**<0.001**	—	0.052	After noon
Honeybee	Solar radiation	**<0.001**	**0.008**	0.078	Before noon
Honeybee	Solar radiation	**<0.001**	**<0.001**	0.27	After noon
Diptera	Ambient temperature	0.321	0.433	0.014	Before noon
Diptera	Ambient temperature	0.215	0.275	0.066	After noon
Diptera	Relative humidity	0.329	0.180	0.035	Before noon
Diptera	Relative humidity	**0.037**	0.329	0.06	After noon
Diptera	Wind velocity	0.180	—	0.016	Before noon
Diptera	Wind velocity	**<0.001**	—	0.077	After noon
Diptera	Solar radiation	**0.019**	0.180	0.056	Before noon
Diptera	Solar radiation	**<0.001**	**<0.001**	0.308	After noon
Solitary bee	Ambient temperature	**<0.001**	0.087	0.176	All day
Solitary bee	Relative humidity	**<0.001**	**0.003**	0.238	All day
Solitary bee	Wind velocity	**0.001**		0.068	All day
Solitary bee	Solar radiation	**<0.001**	**<0.001**	0.331	All day
Lepidoptera	Ambient temperature	0.458	0.760	0.052	Before noon
Lepidoptera	Ambient temperature	0.556	0.556	0.006	After noon
Lepidoptera	Relative humidity	0.204	0.307	0.091	Before noon
Lepidoptera	Relative humidity	0.505	0.556	0.014	After noon
Lepidoptera	Wind velocity	0.760	—	0.002	Before noon
Lepidoptera	Wind velocity	0.630	—	0.004	After noon
Lepidoptera	Solar radiation	0.325	0.448	0.035	Before noon
Lepidoptera	Solar radiation	0.052	0.052	0.13	After noon
Other insects	Ambient temperature	0.434	0.813	0.023	Before noon
Other insects	Ambient temperature	0.973	0.927	0.002	After noon
Other insects	Relative humidity	0.866	0.778	0.003	Before noon
Other insects	Relative humidity	0.813	0.973	0.011	After noon
Other insects	Wind velocity	0.813	—	0.002	Before noon
Other insects	Wind velocity	0.716	—	0.012	After noon
Other insects	Solar radiation	0.501	0.813	0.016	Before noon
Other insects	Solar radiation	0.108	0.08	0.211	After noon

Note

Values (including the Marginal R2‐values) are derived from generalized linear mixed models. Environmental variables' significance was corrected for multiple testing and calculated as *p*‐adjusted. Significant effects at *p* < .05 are presented in bold.

The activity pattern of the solitary bee group was unimodal, showing a positive relationship with ambient temperature and solar radiation. The solitary bee group also showed a negative relationship with relative humidity (Table 3; Table S6). As wind velocity did not include quadratic terms, so the abundance of flower visitors showed only a linear relationship with wind velocity (Figures S7–S10). In contrast, the abundance of lepidopteran and other insects was not significant for all environmental factors (Table 3; Table S6).

After running the marginal R^2^ value, solar radiation (0.180 ± 0.122) was found to be higher than other environmental factors (ambient temperature was 0.095 ± 0.077, relative humidity was 0.085 ± 0.069, and wind velocity was 0.043 ± 0.037) (Table 3). This indicated that solar radiation had the highest fit to explain the variance of flower visitor acitivity.

### 3.3 Effect of habitat on the abundance and activity of flower visitors

Habitat type influenced significantly the abundance of flower visitors (*p* < .05). The abundances of overall flower visitors (*p* < .001), bumblebees (*p* = 0.001), honeybees (*p* = .034), and dipterans (*p* = .05) were higher in meadows than in forests (Table 2; Tables S3–S4; Figure S5). However, the abundances of solitary bees (*p* = .805), lepidopterans (*p* = .434), and other insects (*p* = .171) did not vary between these two habitats (Table 2; Table S3). For most flower‐visiting groups, the overlapping degree was higher than 80% excluding solitary bees and other insects, at less than 80%. The diurnal activity curves of overall flower visitors, bumblebees, honeybees, dipterans, solitary bees, and other insects in the two habitat types showed significant differences (Table 4), but not for lepidopterans with similar diurnal activity patterns regardless of habitat (overlap Δ = 0.801, *p* = .163).

**TABLE 4:** Results of the degree of overlap between the diurnal activity patterns between meadow and forest for different visitor groups across flowering seasons 2018 and 2019 on Yulong Snow Mountain, SW China

Functional groups	Overlap coefficient (Δ)	Null	se‐Null	*p*‐value
Overall	0.908	0.968	0.009	**<0.001**
Bumblebee	0.868	0.935	0.020	**0.003**
Honeybee	0.893	0.958	0.015	**0.002**
Diptera	0.819	0.938	0.022	**<0.001**
Solitary bee	0.771	0.915	0.037	**0.001**
Lepidoptera	0.801	0.862	0.064	0.163
Other insects	0.589	0.830	0.066	**0.001**

Note

Values are derived from nonparametric bootstrapping iteration comparing the activity pattern between two habitats. Significant effects at *p* < .05 are presented in bold.

## 4 DISCUSSION

We found a consistent bimodal pattern of foraging activity of diurnal flower visitors across meadow and forest habitats in six high elevation plant communities. We further showed that this general bimodal activity pattern can be explained at least in part by abiotic environmental factors such as solar radiation. Although relative humidity also helped to explain forager activity patterns, the remaining environmental factors were not relevant in our study. The most likely explanation for this was that we selected only sunny days to do sample sites avoiding extreme weather events.

Most of the previous studies of flower visitor activity focused on a single or few insect species and/or their visits to a focal plant. Most of these studies found that their insects showed a unimodal pattern when visiting flowers (Muniz et al., 2013; Sgolastra et al., 2016; Steen, 2017; Totland, 1994). However, some of these insect species also demonstrated patterns of bimodal activity (Barônio & Torezan‐Silingardi, 2017; Gottlieb et al., 2005) or revealed multiple and daily peaks of flower visitation (Herrera, 1990). Our community‐wide study illustrated a bimodal pattern. Our results are similar to an Arctic flowering community showing a bimodal foraging activity in the course of 1 year (Zoller et al., 2020). In contrast to our findings and the Arctic study, a unimodal activity pattern was also found for all insect groups in ruderal meadow communities in Switzerland (Knop, Gerpe, et al., 2017). Therefore, our findings supplement our ongoing understanding of the dynamics of plant–pollinator interactions and pollination networks at the community level in high elevation environments. The difference is that we suggest that flower visitors are more likely to produce bimodal and multimodal activity patterns in more extreme regions at far higher high elevations and in polar regions.

Furthermore, the overall bimodal activity pattern in this study was consistent across flower visitor groups except for solitary bees, but the exact time among the flower visitor groups' activity peak was similar (Figure 1). Our study area was dominated by polylectic bumblebees, and their activity patterns showed a bimodal distribution. Bumblebee species are typically covered with dense hair, vibrate their thoracic muscles to regulate body temperatures, and are among the largest floral foraging insects in the cooler‐temperate zones (Goulson, 2010). Therefore, they appear well adapted to active foraging in alpine and subalpine habitats representing the most important pollinators in montane regions (Biella, Bogliani, et al., 2017; Egawa & Itino, 2019; Minachilis et al., 2020; Williams et al., 2009). Unimodal activity of solitary bees might be caused by avoiding competition with other insect groups (including bumblebees) in alpine regions, or these smaller bees may employ the unimodal pattern to increase their body temperatures, so they can continue to forage under temperatures at higher elevations (Willmer & Stone, 2004).

Interestingly, in our study temperature was not a good predictor to explain pollinator activity. This is in contrast with previous studies that found temperature to be the main predictor of pollinator activity (Knop, Gerpe, et al., 2017; Kühsel & Blüthgen, 2015; Zoller et al., 2020). Temperatures were relatively low (9.2–21.2°C) at our sites due to their high elevation. The most likely reason why we found this contrasting pattern was the interaction between solar radiation and temperature. The highest solar radiation at noon should increase body temperature of insects at a rapid rate (Cena & Clark, 1972; Corbet et al., 2020). As solar radiation increases the body temperature of an insect at a certain level, it becomes in danger of overheating, so foraging activity must decrease. Several insect species show adaptions to high solar radiation (e.g., UV radiation) at high elevations (Birrell et al., 2020; Hodkinson, 2005).

Consistent with the expectation mentioned above, in our study solar radiation showed a positive relation to insect activity until a certain threshold was reached. This relationship most likely determined the bimodal distribution of visits. At high elevations, solar radiation (e.g., UV radiation) is an important driver of species performance and behavior. In our study, a decrease in activity usually occurred from 1:00 to 2:00 p.m. During this time period, the solar radiation value was higher than 450 μmol·m^−2^·s^−1^ on 9 out of the 14 study days. Beyond this solar radiation threshold, the activity of members of our flower visitor community decreased with the exception of bumblebees and dipterans (Figure 2), and this explains the decrease in general activity at midday. Our results indicate that high solar radiation may overheat insect bodies causing declines in foraging as in other studies (Barônio & Torezan‐Silingardi, 2017; Bellusci & Marques, 2001; Herrera, 1990). The deviation of a bumblebee's lack of a response to peak solar radiation is most likely explained by their ectothermic mode of thermoregulation preventing them from overheating (Heinrich, 1979). Also, sisters in the same colony show multiple flight bouts for collecting floral resources during the same day, both allowing the same colony to continue foraging even during peaks of solar radiation.

Higher solar radiation increases both insect body temperature (Cena & Clark, 1972; Corbet et al., 2020) and the temperature of flowers (Corbet et al., 2020). It has been shown that elevated temperatures, caused by solar radiation, evaporate plant floral nectars making it more difficult for foragers to extract remaining nutrients. This reduces visitation rates and decreases pollination success lowering rates of sexual reproduction in plants (Corbet et al., 2020; Scaven & Rafferty, 2013). Therefore, temporal patterns of pollinator nectar‐feeding and nectar production may coincide. However, a previous experiment with *Anemone rivularis* conducted in the same area as our study revealed that at midday, when the ambient temperature was highest and the radiation strongest, the internal flower temperature of the plant was lower than the ambient temperature. Such an experiment proves that plants can regulate microenvironments inside their flowers to avoid damage by higher temperatures and radiation (Zhang et al., 2010) possibly limiting their effects on nectar production. Matching of nectar resource availability with insect flower‐visiting schedules warrants further investigations in our Himalayan communities.

There was a generally high degree of activity overlap between the meadow and the forest habitats (>80% for most flower visitor groups), but the activity curves differed somewhat between forest and meadow (Table 4). For example, the two flower visitor groups with highest abundance, bumblebees and honeybees, visited flowers during the afternoons in the meadow more often than in the forest (Figure 1b,c). This may be due to the fact that both *Apis* and *Bombus* species are generalized floral foragers reducing competition within members of the same groups and between different visitor groups, thus sustaining their high abundance. That is, although the floral resources in alpine meadows are highly frequented, they can still support higher visitation rates compared to forest habitats. However, the habitat effect proved the opposite for less abundant visitor groups (dipterans, solitary bees, lepidopterans, and other insects). They showed a higher abundance in the forest than in the meadow during afternoons (Figure 1d–g). This asymmetry in habitat transition between different flower visitor groups suggests that alterations in the availability of floral resources may differentially affect habitat and foraging preferences across these flower visitor groups. In the afternoon, bees become more abundant in the meadow, forcing other insects to switch to floral resources in forests to avoiding competition via temporal and/or spatial displacement.

## 5 CONCLUSION

In conclusion, this appears to be the first study to show temporal foraging activity patterns in pollination communities at their highest (Himalayan) elevations. We found bimodal activities of some flower visitors in this Himalayan environment. We highlighted a consistency in bimodal visitation patterns between meadow and forest habitats, while showing differences in foraging peaks of flower visitor groups between both habitats. This suggests microsite variability. Finally, we suggest that the general activity decline in foraging activity at midday is most likely explained by increased solar radiation at higher elevations. Clearly, further research is needed to clarify the impact of solar radiation on alpine plants and their flower visitors. Mountain regions are characterized by steep changes in climatic conditions and high microsite heterogeneity. Thus, future research should investigate the spatio‐temporal dynamics of plant–visitor interactions at different elevations and in variable microhabitat niches to better understand variation in temporal and spatial variation in insect pollination ultimately driving annual rates of plant reproduction and long‐term floral evolution. Understanding the climatic factors influencing plant and visitor interactions in montane environments is important to predict possible responses to ongoing climatic changes.

## CONFLICT OF INTEREST

The authors declare no competing interests.

## AUTHOR CONTRIBUTIONS

**Xin Xu:** Formal analysis (equal); Investigation (lead); Software (equal); Visualization (equal); Writing‐original draft (lead); Writing‐review & editing (equal). **Zong‐Xin Ren:** Conceptualization (equal); Methodology (equal); Writing‐review & editing (equal). **Judith Trunschke:** Formal analysis (equal); Software (equal); Visualization (equal); Writing‐review & editing (equal). **Jonas Kuppler:** Formal analysis (equal); Software (equal); Visualization (equal); Writing‐review & editing (equal). **Yan‐Hui Zhao:** Investigation (supporting); Writing‐review & editing (equal). **Eva Knop:** Conceptualization (equal); Methodology (equal); Writing‐review & editing (equal). **Hong Wang:** Conceptualization (equal); Methodology (equal); Writing‐review & editing (equal).

## Supporting information

Appendix S1

## Data Availability

The dataset has been deposited in the Dryad and is accessible under https://doi.org/10.5061/dryad.1vhhmgqtf.
