# Supplementary material for: Bimodal activity of diurnal flower visitation at high elevation
Source: Ecol Evol. 2021 Sep 2;11(19):13487–500. doi: 10.1002/ece3.8074 (PMC8495799; doi:10.1002/ece3.8074)
Supplement: Supplementary file 1 — Appendix S1 [file ECE3-11-13487-s001.docx]

**Appendix S1**

**Bimodal activity of** **diurnal flo****wer** **visitation at high elevation**

Xin Xu^1, 2^, Zong-Xin Ren^1, 6, *^, Judith Trunschke^1^, Jonas Kuppler^3^, Yan-Hui Zhao^1^, Eva Knop^4, 5^, Hong Wang^1, 2, *^

**Tables:**

Table S1. Coordinates and elevation of the six study transects.

| Transects | Latitude | Longitude | Elevation(m) |
| --- | --- | --- | --- |
| Meadow-1 | 27°00′09″ | 100°10′57″ | 3240 |
| Forest-1 | 27°00′09″ | 100°10′57″ | 3240 |
| Meadow-2 | 26°59′21″ | 100°10′27″ | 3200 |
| Forest-2 | 26°59′21″ | 100°10′27″ | 3200 |
| Meadow-3 | 26°58′42″ | 100°10′46″ | 3170 |
| Forest-3 | 26°58′42″ | 100°10′46″ | 3170 |

Table S2. Result of the degree of overlap in the diurnal activity patterns between the different flower visitor groups in meadow and forest in the 2018 and 2019 flowering seasons on Yulong Mountain, SW China.

| Transect  habitat | Functional  group A | Functional  group B | Overlap  coefficient (Δ) | Null | seNull | P-value |
| --- | --- | --- | --- | --- | --- | --- |
| Meadow | Bumblebee | Honeybee | 0.857 | 0.971 | 0.009 | **< 0.001** |
|  |  | Diptera | 0.748 | 0.964 | 0.011 | **< 0.001** |
|  |  | Solitary bee | 0.741 | 0.919 | 0.025 | **< 0.001** |
|  |  | Lepidoptera | 0.688 | 0.911 | 0.027 | **< 0.001** |
|  |  | Other insects | 0.578 | 0.912 | 0.028 | **< 0.001** |
|  | Honeybee | Diptera | 0.887 | 0.964 | 0.012 | **< 0.001** |
|  |  | Solitary bee | 0.881 | 0.927 | 0.025 | 0.050 |
|  |  | Lepidoptera | 0.813 | 0.918 | 0.027 | **0.001** |
|  |  | Other insects | 0.708 | 0.918 | 0.027 | **< 0.001** |
|  | Diptera | Solitary bee | 0.892 | 0.928 | 0.025 | 0.083 |
|  |  | Lepidoptera | 0.904 | 0.922 | 0.027 | 0.233 |
|  |  | Other insects | 0.822 | 0.924 | 0.028 | **0.004** |
|  | Solitary bee | Lepidoptera | 0.852 | 0.919 | 0.035 | 0.051 |
|  |  | Other insects | 0.765 | 0.920 | 0.034 | **< 0.001** |
|  | Lepidoptera | Other insects | 0.845 | 0.925 | 0.035 | **0.022** |
| Forest | Bumblebee | Honeybee | 0.864 | 0.950 | 0.022 | **0.003** |
|  |  | Diptera | 0.822 | 0.938 | 0.026 | **0.001** |
|  |  | Solitary bee | 0.738 | 0.919 | 0.035 | **0.001** |
|  |  | Lepidoptera | 0.873 | 0.862 | 0.060 | 0.507 |
|  |  | Other insects | 0.920 | 0.849 | 0.066 | 0.876 |
|  | Honeybee | Diptera | 0.841 | 0.938 | 0.023 | **< 0.001** |
|  |  | Solitary bee | 0.743 | 0.913 | 0.033 | **0.001** |
|  |  | Lepidoptera | 0.884 | 0.839 | 0.056 | 0.773 |
|  |  | Other insects | 0.797 | 0.823 | 0.064 | 0.316 |
|  | Diptera | Solitary bee | 0.891 | 0.911 | 0.035 | 0.234 |
|  |  | Lepidoptera | 0.941 | 0.847 | 0.058 | 0.979 |
|  |  | Other insects | 0.792 | 0.830 | 0.064 | 0.258 |
|  | Solitary bee | Lepidoptera | 0.824 | 0.843 | 0.062 | 0.339 |
|  |  | Other insects | 0.720 | 0.825 | 0.068 | 0.084 |
|  | Lepidoptera | Other insects | 0.845 | 0.840 | 0.087 | 0.442 |

Note: Results are derived from non-parametric bootstrapping iterations comparing the activity pattern among functional groups. Significant effects at P < 0.05 are presented in bold.

Table S3. Result of the conditional models.

| Parameter | Estimate | SE | z value | Pr>\|z\| |
| --- | --- | --- | --- | --- |
| **Overall** |  |  |  |  |
| Intercept | 1.125 | 0.153 | 7.364 | **< 0.001** |
| Relative humidity | -0.132 | 0.083 | -1.584 | 0.113 |
| Relative humidity^2^ | -0.134 | 0.039 | -3.399 | **0.001** |
| Wind velocity | 0.104 | 0.076 | 1.370 | 0.171 |
| Solar radiation | 0.433 | 0.117 | 3.697 | **< 0.001** |
| Solar radiation^2^ | -0.106 | 0.054 | -1.962 | **0.050** |
| Habitat Meadow | 1.335 | 0.183 | 7.296 | **< 0.001** |
| **Bumblebee** |  |  |  |  |
| Intercept | -0.437 | 0.448 | -0.975 | 0.330 |
| Relative humidity | -0.325 | 0.101 | -3.215 | **0.001** |
| Relative humidity^2^ | -0.096 | 0.048 | -1.982 | **0.048** |
| Wind velocity | 0.058 | 0.087 | 0.675 | 0.500 |
| Solar radiation | 0.287 | 0.139 | 2.066 | **0.039** |
| Solar radiation^2^ | -0.025 | 0.061 | -0.411 | 0.681 |
| Habitat Meadow | 1.641 | 0.499 | 3.290 | **0.001** |
| **Honeybee** |  |  |  |  |
| Intercept | -0.408 | 0.371 | -1.100 | 0.271 |
| Relative humidity | 0.070 | 0.118 | 0.589 | 0.556 |
| Relative humidity^2^ | -0.055 | 0.065 | -0.851 | 0.395 |
| Wind velocity | 0.222 | 0.117 | 1.890 | 0.059 |
| Solar radiation | 1.509 | 0.150 | 10.092 | **< 0.001** |
| Solar radiation^2^ | -0.526 | 0.072 | -7.314 | **< 0.001** |
| Habitat Meadow | 1.053 | 0.497 | 2.118 | **0.034** |
| **Diptera** |  |  |  |  |
| Intercept | -0.336 | 0.300 | -1.118 | 0.263 |
| Relative humidity | 0.183 | 0.141 | 1.299 | 0.194 |
| Relative humidity^2^ | -0.048 | 0.078 | -0.618 | 0.537 |
| Wind velocity | 0.066 | 0.144 | 0.462 | 0.644 |
| Solar radiation | 0.486 | 0.230 | 2.111 | **0.035** |
| Solar radiation^2^ | -0.112 | 0.109 | -1.029 | 0.304 |
| Habitat Meadow | 0.905 | 0.319 | 2.838 | **0.005** |
| **Solitary bee** |  |  |  |  |
| Intercept | -1.508 | 0.480 | -3.142 | **0.002** |
| Relative humidity | -0.367 | 0.246 | -1.494 | 0.135 |
| Relative humidity^2^ | -0.108 | 0.161 | -0.673 | 0.501 |
| Wind velocity | -0.097 | 0.220 | -0.439 | 0.661 |
| Solar radiation | 1.196 | 0.280 | 4.265 | **< 0.001** |
| Solar radiation^2^ | -0.468 | 0.176 | -2.659 | **0.008** |
| Habitat Meadow | 0.156 | 0.629 | 0.248 | 0.805 |
| **Lepidoptera** |  |  |  |  |
| Intercept | -1.190 | 0.478 | -2.487 | **0.013** |
| Relative humidity | 0.238 | 0.232 | 1.023 | 0.306 |
| Relative humidity^2^ | -0.065 | 0.172 | -0.376 | 0.707 |
| Wind velocity | 0.059 | 0.224 | 0.265 | 0.791 |
| Solar radiation | 0.841 | 0.272 | 3.088 | **0.002** |
| Solar radiation^2^ | -0.630 | 0.215 | -2.930 | **0.003** |
| Habitat Meadow | 0.341 | 0.436 | 0.783 | 0.434 |
| **Other insects** |  |  |  |  |
| Intercept | -2.002 | 0.803 | -2.494 | **0.013** |
| Relative humidity | 0.304 | 0.355 | 0.857 | 0.391 |
| Relative humidity^2^ | 0.325 | 0.263 | 1.237 | 0.216 |
| Wind velocity | -0.032 | 0.468 | -0.069 | 0.945 |
| Solar radiation | 1.518 | 0.537 | 2.826 | **0.005** |
| Solar radiation^2^ | -1.150 | 0.347 | -3.319 | **0.001** |
| Habitat Meadow | 1.223 | 0.894 | 1.368 | 0.171 |

Note: Significant effects at P < 0.05 are presented in bold.

Table S4. Result of the zero-inflation models.

| Parameter | Estimate | SE | z value | Pr>\|z\| |
| --- | --- | --- | --- | --- |
| **Overall** |  |  |  |  |
| Intercept | -0.512 | 0.239 | -2.138 | **0.033** |
| Solar radiation | -2.106 | 0.187 | -11.259 | **< 0.001** |
| Solar radiation^2 | 0.810 | 0.098 | 8.227 | **< 0.001** |
| Habitat Meadow | -0.893 | 0.231 | -3.865 | **< 0.001** |
| **Bumblebee** |  |  |  |  |
| Intercept | -1.906 | 0.421 | -4.531 | **< 0.001** |
| Solar radiation | -2.615 | 0.432 | -6.047 | **< 0.001** |
| Solar radiation^2 | 1.103 | 0.206 | 5.356 | **< 0.001** |
| **Diptera** |  |  |  |  |
| Intercept | -3.217 | 1.027 | -3.133 | **0.002** |
| Solar radiation | -5.398 | 1.223 | -4.415 | **< 0.001** |

Note: Significant effects at P < 0.05 are presented in bold.

Table S5. Results of the effect of environmental variables (ambient temperature instead of solar radiation) on the abundance of flower visitors in two flowering seasons on Yulong Snow Mountain, SW China. Values are derived from generalized linear mixed models and significance was assessed with Type III Wald Chi-square tests. Significant effects at P < 0.05 are presented in bold.

| Parameter | Chi-sq. | Df | P>Chi-sq. |
| --- | --- | --- | --- |
| **Overall** |  |  |  |
| Intercept | 70.1994 | 1 | **< 0.001** |
| Relative humidity | 3.3282 | 1 | 0.068 |
| Relative humidity^2^ | 9.123 | 1 | **0.003** |
| Wind velocity | 13.5638 | 1 | **< 0.001** |
| Ambient temperature | 0.0161 | 1 | 0.899 |
| Ambient temperature^2^ | 0.3492 | 1 | 0.555 |
| Habitat type | 57.2903 | 1 | **< 0.001** |
| **Bumblebee** |  |  |  |
| Intercept | 1.3146 | 1 | 0.252 |
| Relative humidity | 0.4295 | 1 | 0.512 |
| Relative humidity^2^ | 4.936 | 1 | **0.026** |
| Wind velocity | 6.832 | 1 | **0.009** |
| Ambient temperature | 3.0895 | 1 | 0.079 |
| Ambient temperature^2^ | 2.506 | 1 | 0.113 |
| Habitat type | 10.0777 | 1 | **0.002** |
| **Honeybee** |  |  |  |
| Intercept | 0.9875 | 1 | 0.320 |
| Relative humidity | 0.4826 | 1 | 0.487 |
| Relative humidity^2^ | 3.6681 | 1 | 0.055 |
| Wind velocity | 29.9294 | 1 | **< 0.001** |
| Ambient temperature | 4.1667 | 1 | **0.041** |
| Ambient temperature^2^ | 6.2389 | 1 | **0.013** |
| Habitat type | 5.355 | 1 | **0.021** |
| **Diptera** |  |  |  |
| Intercept | 0.1198 | 1 | 0.729 |
| Relative humidity | 0.1139 | 1 | 0.736 |
| Relative humidity^2^ | 5.8081 | 1 | **0.016** |
| Wind velocity | 10.4142 | 1 | **0.001** |
| Ambient temperature | 0.0131 | 1 | 0.909 |
| Ambient temperature^2^ | 0.0459 | 1 | 0.830 |
| Habitat type | 8.384 | 1 | **0.004** |
| **Solitary bee** |  |  |  |
| Intercept | 11.2929 | 1 | **0.001** |
| Relative humidity | 10.4903 | 1 | **0.001** |
| Relative humidity^2^ | 9.8814 | 1 | **0.002** |
| Wind velocity | 1.8718 | 1 | 0.171 |
| Ambient temperature | 2.0536 | 1 | 0.152 |
| Ambient temperature^2^ | 2.6606 | 1 | 0.103 |
| Habitat type | 0.0716 | 1 | 0.789 |
| **Lepidoptera** |  |  |  |
| Intercept | 10.1755 | 1 | **0.001** |
| Relative humidity | 0.269 | 1 | 0.604 |
| Relative humidity^2^ | 2.5314 | 1 | 0.112 |
| Wind velocity | 1.387 | 1 | 0.239 |
| Ambient temperature | 0.1355 | 1 | 0.713 |
| Ambient temperature^2^ | 0.1145 | 1 | 0.735 |
| Habitat type | 1.0582 | 1 | 0.304 |
| **Other insects** |  |  |  |
| Intercept | 7.6069 | 1 | **0.006** |
| Relative humidity | 0.057 | 1 | 0.811 |
| Relative humidity^2^ | 1.7884 | 1 | 0.181 |
| Wind velocity | 1.5595 | 1 | 0.212 |
| Ambient temperature | 0.0003 | 1 | 0.986 |
| Ambient temperature^2^ | 3.6342 | 1 | 0.057 |
| Habitat type | 1.5489 | 1 | 0.213 |

Table S6. Results of the effect of separately the environmental variables associated with each abundance peak independently. Values are derived from generalized linear mixed models and significance was assessed with Type III Wald Chi-square tests. Moreover, environmental variables’ significance was corrected for multiple testing and calculated as P-adjust. Significant effects at P < 0.05 are presented in bold.

|  | Estimate | Chisq | Df | Pr(>Chisq) | P.adjust | Time of the day |
| --- | --- | --- | --- | --- | --- | --- |
| Overall |  |  |  |  |  |  |
| Intercept | 1.881 | 15.832 | 1 | **< 0.001** | **< 0.001** | Before noon |
| Ambient temperature | 0.440 | 16.521 | 1 | **< 0.001** | **< 0.001** | Before noon |
| Ambient temperature^2^ | -0.523 | 18.228 | 1 | **< 0.001** | **< 0.001** | Before noon |
| Intercept | 0.723 | 2.478 | 1 | 0.115 | 0.115 | After noon |
| Ambient temperature | 1.193 | 53.244 | 1 | **< 0.001** | **< 0.001** | After noon |
| Ambient temperature^2^ | -0.150 | 3.553 | 1 | 0.059 | 0.062 | After noon |
| Intercept | 1.820 | 15.154 | 1 | **< 0.001** | **< 0.001** | Before noon |
| Relative humidity | -0.426 | 19.715 | 1 | **< 0.001** | **< 0.001** | Before noon |
| Relative humidity^2^ | -0.336 | 15.220 | 1 | **< 0.001** | **< 0.001** | Before noon |
| Intercept | 1.032 | 6.156 | 1 | **0.013** | **0.015** | After noon |
| Relative humidity | -0.889 | 48.897 | 1 | **< 0.001** | **< 0.001** | After noon |
| Relative humidity^2^ | -0.146 | 5.704 | 1 | **0.017** | **0.019** | After noon |
| Intercept | 1.597 | 12.859 | 1 | **< 0.001** | **< 0.001** | Before noon |
| Wind velocity | 0.337 | 15.891 | 1 | **< 0.001** | **< 0.001** | Before noon |
| Intercept | 1.264 | 11.190 | 1 | **0.001** | **0.001** | After noon |
| Wind velocity | 0.474 | 40.081 | 1 | **< 0.001** | **< 0.001** | After noon |
| Intercept | 1.551 | 10.491 | 1 | **0.001** | **0.002** | Before noon |
| Solar radiation | 0.805 | 41.325 | 1 | **< 0.001** | **< 0.001** | Before noon |
| Solar radiation^2^ | -0.350 | 16.823 | 1 | **< 0.001** | **< 0.001** | Before noon |
| Intercept | 1.280 | 7.687 | 1 | **0.006** | **0.007** | After noon |
| Solar radiation | 1.386 | 173.384 | 1 | **< 0.001** | **< 0.001** | After noon |
| Solar radiation^2^ | -0.506 | 73.333 | 1 | **< 0.001** | **< 0.001** | After noon |
| Bumblebee |  |  |  |  |  |  |
| Intercept | 0.539 | 0.762 | 1 | 0.383 | 0.526 | Before noon |
| Ambient temperature | 0.746 | 25.665 | 1 | **< 0.001** | **< 0.001** | Before noon |
| Ambient temperature^2^ | -0.528 | 8.391 | 1 | **0.004** | **0.007** | Before noon |
| Intercept | -0.430 | 0.473 | 1 | 0.492 | 0.594 | After noon |
| Ambient temperature | 1.792 | 66.307 | 1 | **< 0.001** | **< 0.001** | After noon |
| Ambient temperature^2^ | -0.333 | 7.347 | 1 | **0.007** | **0.011** | After noon |
| Intercept | 0.464 | 0.578 | 1 | 0.447 | 0.579 | Before noon |
| Relative humidity | -0.716 | 29.262 | 1 | **< 0.001** | **< 0.001** | Before noon |
| Relative humidity^2^ | -0.329 | 10.179 | 1 | **0.001** | **0.003** | Before noon |
| Intercept | -0.028 | 0.002 | 1 | 0.961 | 0.961 | After noon |
| Relative humidity | -1.091 | 53.139 | 1 | **< 0.001** | **< 0.001** | After noon |
| Relative humidity^2^ | -0.149 | 3.954 | 1 | **0.047** | 0.073 | After noon |
| Intercept | 0.263 | 0.208 | 1 | 0.648 | 0.713 | Before noon |
| Wind velocity | 0.532 | 19.266 | 1 | **< 0.001** | **< 0.001** | Before noon |
| Intercept | 0.457 | 0.883 | 1 | 0.347 | 0.509 | After noon |
| Wind velocity | 0.549 | 32.952 | 1 | **< 0.001** | **< 0.001** | After noon |
| Intercept | 0.078 | 0.016 | 1 | 0.899 | 0.942 | Before noon |
| Solar radiation | 1.029 | 34.699 | 1 | **< 0.001** | **< 0.001** | Before noon |
| Solar radiation^2^ | -0.335 | 8.683 | 1 | **0.003** | **0.006** | Before noon |
| Intercept | 0.373 | 0.428 | 1 | 0.513 | 0.594 | After noon |
| Solar radiation | 1.375 | 109.255 | 1 | **< 0.001** | **< 0.001** | After noon |
| Solar radiation^2^ | -0.484 | 42.420 | 1 | **< 0.001** | **< 0.001** | After noon |
| Honeybee |  |  |  |  |  |  |
| Intercept | 0.880 | 8.341 | 1 | **0.004** | **0.009** | Before noon |
| Ambient temperature | 0.243 | 3.234 | 1 | 0.072 | 0.099 | Before noon |
| Ambient temperature^2^ | -0.475 | 8.658 | 1 | **0.003** | **0.009** | Before noon |
| Intercept | -0.217 | 0.367 | 1 | 0.545 | 0.625 | After noon |
| Ambient temperature | 0.958 | 21.811 | 1 | **< 0.001** | **< 0.001** | After noon |
| Ambient temperature^2^ | -0.136 | 2.256 | 1 | 0.133 | 0.163 | After noon |
| Intercept | 0.830 | 7.770 | 1 | **0.005** | **0.012** | Before noon |
| Relative humidity | -0.205 | 2.445 | 1 | 0.118 | 0.153 | Before noon |
| Relative humidity^2^ | -0.304 | 6.104 | 1 | **0.013** | **0.025** | Before noon |
| Intercept | 0.049 | 0.022 | 1 | 0.882 | 0.882 | After noon |
| Relative humidity | -0.747 | 21.256 | 1 | **< 0.001** | **< 0.001** | After noon |
| Relative humidity^2^ | -0.173 | 4.804 | 1 | **0.028** | **0.042** | After noon |
| Intercept | 0.690 | 6.652 | 1 | **0.01** | **0.020** | Before noon |
| Wind velocity | 0.239 | 5.501 | 1 | **0.019** | **0.032** | Before noon |
| Intercept | 0.177 | 0.325 | 1 | 0.568 | 0.625 | After noon |
| Wind velocity | 0.362 | 19.172 | 1 | **< 0.001** | **< 0.001** | After noon |
| Intercept | 0.682 | 5.072 | 1 | **0.024** | **0.038** | Before noon |
| Solar radiation | 0.676 | 16.917 | 1 | **< 0.001** | **< 0.001** | Before noon |
| Solar radiation^2^ | -0.391 | 9.178 | 1 | **0.002** | **0.008** | Before noon |
| Intercept | 0.162 | 0.215 | 1 | 0.643 | 0.674 | After noon |
| Solar radiation | 1.184 | 77.825 | 1 | **< 0.001** | **< 0.001** | After noon |
| Solar radiation^2^ | -0.396 | 29.105 | 1 | **< 0.001** | **< 0.001** | After noon |
| Diptera |  |  |  |  |  |  |
| Intercept | 0.498 | 2.470 | 1 | 0.116 | 0.215 | Before noon |
| Ambient temperature | 0.157 | 1.180 | 1 | 0.277 | 0.321 | Before noon |
| Ambient temperature^2^ | -0.131 | 0.615 | 1 | 0.433 | 0.433 | Before noon |
| Intercept | -0.415 | 3.944 | 1 | **0.047** | 0.173 | After noon |
| Ambient temperature | 0.323 | 2.327 | 1 | 0.127 | 0.215 | After noon |
| Ambient temperature^2^ | 0.119 | 1.703 | 1 | 0.192 | 0.275 | After noon |
| Intercept | 0.565 | 3.386 | 1 | 0.066 | 0.180 | Before noon |
| Relative humidity | -0.155 | 1.064 | 1 | 0.302 | 0.329 | Before noon |
| Relative humidity^2^ | -0.261 | 3.432 | 1 | 0.064 | 0.180 | Before noon |
| Intercept | -0.287 | 2.428 | 1 | 0.119 | 0.215 | After noon |
| Relative humidity | -0.518 | 6.953 | 1 | **0.008** | **0.037** | After noon |
| Relative humidity^2^ | -0.092 | 1.015 | 1 | 0.314 | 0.329 | After noon |
| Intercept | 0.401 | 1.640 | 1 | 0.2 | 0.275 | Before noon |
| Wind velocity | 0.215 | 3.202 | 1 | 0.074 | 0.180 | Before noon |
| Intercept | -0.321 | 1.935 | 1 | 0.164 | 0.258 | After noon |
| Wind velocity | 0.435 | 18.462 | 1 | **< 0.001** | **< 0.001** | After noon |
| Intercept | 0.378 | 1.290 | 1 | 0.256 | 0.313 | Before noon |
| Solar radiation | 0.549 | 8.508 | 1 | **0.004** | **0.019** | Before noon |
| Solar radiation^2^ | -0.270 | 3.029 | 1 | 0.082 | 0.180 | Before noon |
| Intercept | -0.337 | 1.555 | 1 | 0.212 | 0.275 | After noon |
| Solar radiation | 1.277 | 59.315 | 1 | **< 0.001** | **< 0.001** | After noon |
| Solar radiation^2^ | -0.463 | 25.798 | 1 | **< 0.001** | **< 0.001** | After noon |
| Solitary bee |  |  |  |  |  |  |
| Intercept | -1.284 | 20.322 | 1 | **< 0.001** | **< 0.001** | All day |
| Ambient temperature | 0.676 | 17.155 | 1 | **< 0.001** | **< 0.001** | All day |
| Ambient temperature^2^ | -0.186 | 2.927 | 1 | 0.087 | 0.087 | All day |
| Intercept | -1.173 | 20.367 | 1 | **< 0.001** | **< 0.001** | All day |
| Relative humidity | -0.876 | 21.891 | 1 | **< 0.001** | **< 0.001** | All day |
| Relative humidity^2^ | -0.377 | 8.784 | 1 | **0.003** | **0.003** | All day |
| Intercept | -1.360 | 22.311 | 1 | **< 0.001** | **< 0.001** | All day |
| Wind velocity | 0.422 | 11.839 | 1 | **0.001** | **0.001** | All day |
| Intercept | -1.319 | 17.293 | 1 | **< 0.001** | **< 0.001** | All day |
| Solar radiation | 1.233 | 36.811 | 1 | **< 0.001** | **< 0.001** | All day |
| Solar radiation^2^ | -0.475 | 12.951 | 1 | **< 0.001** | **< 0.001** | All day |
| Lepidoptera |  |  |  |  |  |  |
| Intercept | -0.875 | 5.746 | 1 | **0.017** | 0.052 | Before noon |
| Ambient temperature | -0.367 | 1.021 | 1 | 0.312 | 0.458 | Before noon |
| Ambient temperature^2^ | 0.102 | 0.104 | 1 | 0.747 | 0.760 | Before noon |
| Intercept | -1.587 | 48.653 | 1 | **< 0.001** | **< 0.001** | After noon |
| Ambient temperature | -0.227 | 0.499 | 1 | 0.48 | 0.556 | After noon |
| Ambient temperature^2^ | 0.136 | 0.561 | 1 | 0.454 | 0.556 | After noon |
| Intercept | -0.563 | 2.512 | 1 | 0.113 | 0.226 | Before noon |
| Relative humidity | 0.952 | 2.825 | 1 | 0.093 | 0.204 | Before noon |
| Relative humidity^2^ | -0.970 | 1.906 | 1 | 0.167 | 0.307 | Before noon |
| Intercept | -1.617 | 47.285 | 1 | **< 0.001** | **< 0.001** | After noon |
| Relative humidity | -0.344 | 0.813 | 1 | 0.367 | 0.505 | After noon |
| Relative humidity^2^ | -0.154 | 0.498 | 1 | 0.48 | 0.556 | After noon |
| Intercept | -0.701 | 4.244 | 1 | **0.039** | 0.096 | Before noon |
| Wind velocity | -0.069 | 0.094 | 1 | 0.76 | 0.760 | Before noon |
| Intercept | -1.615 | 59.203 | 1 | **< 0.001** | **< 0.001** | After noon |
| Wind velocity | 0.103 | 0.318 | 1 | 0.573 | 0.630 | After noon |
| Intercept | -0.828 | 5.105 | 1 | **0.024** | 0.066 | Before noon |
| Solar radiation | -0.456 | 1.701 | 1 | 0.192 | 0.325 | Before noon |
| Solar radiation^2^ | 0.390 | 1.142 | 1 | 0.285 | 0.448 | Before noon |
| Intercept | -1.240 | 24.238 | 1 | **< 0.001** | **< 0.001** | After noon |
| Solar radiation | 0.685 | 6.005 | 1 | **0.014** | 0.052 | After noon |
| Solar radiation^2^ | -0.560 | 6.281 | 1 | **0.012** | 0.052 | After noon |
| Other insects |  |  |  |  |  |  |
| Intercept | -1.723 | 2.093 | 1 | 0.148 | 0.391 | Before noon |
| Ambient temperature | -0.624 | 1.525 | 1 | 0.217 | 0.434 | Before noon |
| Ambient temperature^2^ | -0.255 | 0.335 | 1 | 0.563 | 0.813 | Before noon |
| Intercept | -1.346 | 12.623 | 1 | **< 0.001** | **0.003** | After noon |
| Ambient temperature | 0.017 | 0.001 | 1 | 0.973 | 0.973 | After noon |
| Ambient temperature^2^ | -0.065 | 0.039 | 1 | 0.843 | 0.927 | After noon |
| Intercept | -1.843 | 2.353 | 1 | 0.125 | 0.391 | Before noon |
| Relative humidity | -0.212 | 0.103 | 1 | 0.748 | 0.866 | Before noon |
| Relative humidity^2^ | 0.398 | 0.465 | 1 | 0.495 | 0.778 | Before noon |
| Intercept | -1.527 | 15.182 | 1 | **< 0.001** | **0.001** | After noon |
| Relative humidity | -0.267 | 0.213 | 1 | 0.645 | 0.813 | After noon |
| Relative humidity^2^ | -0.024 | 0.006 | 1 | 0.94 | 0.973 | After noon |
| Intercept | -1.610 | 1.975 | 1 | 0.16 | 0.391 | Before noon |
| Wind velocity | 0.149 | 0.187 | 1 | 0.666 | 0.813 | Before noon |
| Intercept | -1.469 | 17.396 | 1 | **< 0.001** | **0.001** | After noon |
| Wind velocity | 0.211 | 0.642 | 1 | 0.423 | 0.716 | After noon |
| Intercept | -1.538 | 1.724 | 1 | 0.189 | 0.416 | Before noon |
| Solar radiation | -0.292 | 1.201 | 1 | 0.273 | 0.501 | Before noon |
| Solar radiation^2^ | -0.184 | 0.220 | 1 | 0.639 | 0.813 | Before noon |
| Intercept | -0.916 | 5.784 | 1 | **0.016** | 0.080 | After noon |
| Solar radiation | 0.994 | 4.749 | 1 | **0.029** | 0.108 | After noon |
| Solar radiation^2^ | -0.887 | 5.589 | 1 | **0.018** | 0.080 | After noon |

**Figures:**


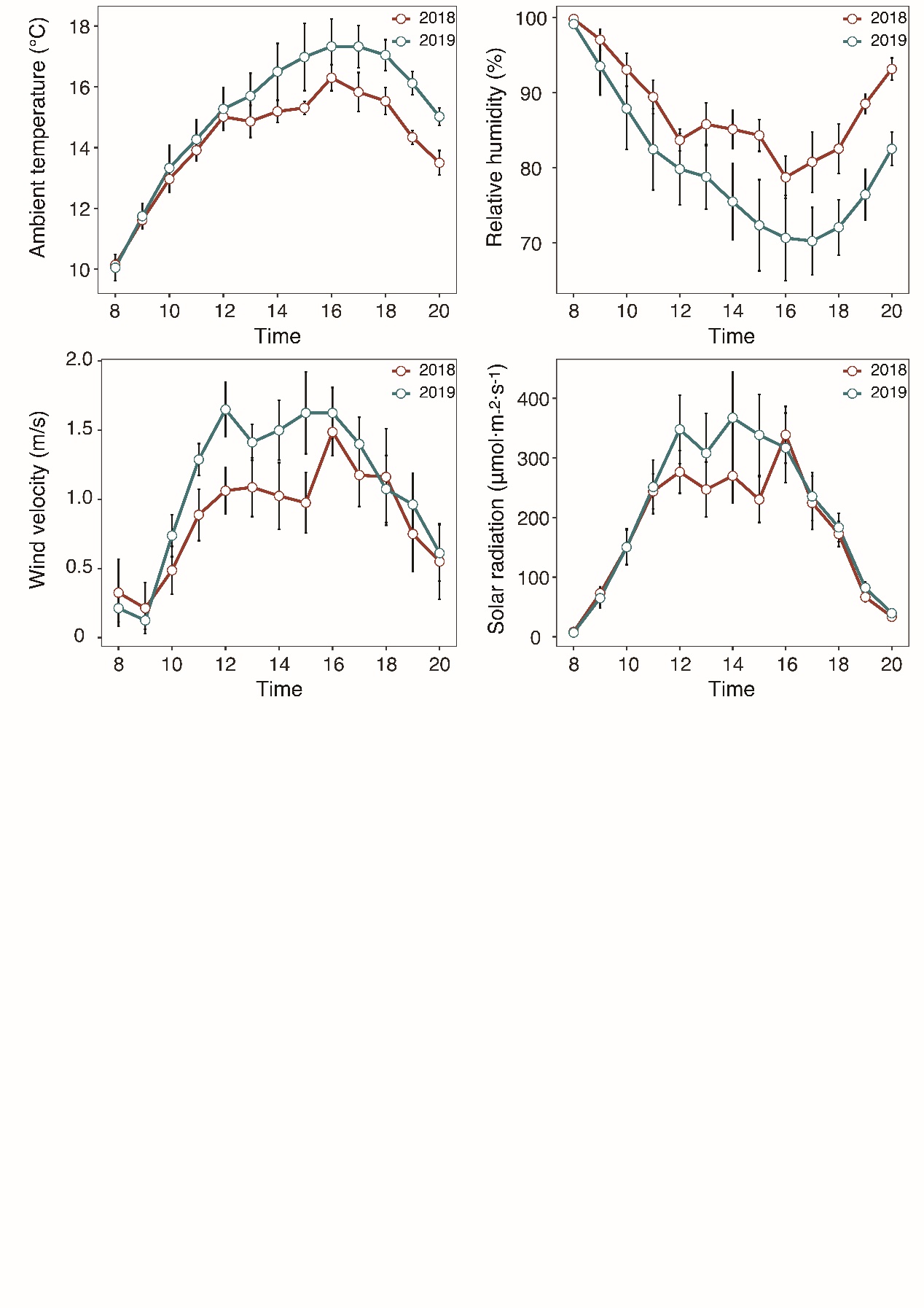


Fig. S1. Temporal variation in temperature (a), relative humidity (b), wind velocity (c) and solar radiation (d) during the sampling days in 2018 (red) and 2019 (green). The point and bar indicate the mean ± SE of sampling days across two years.


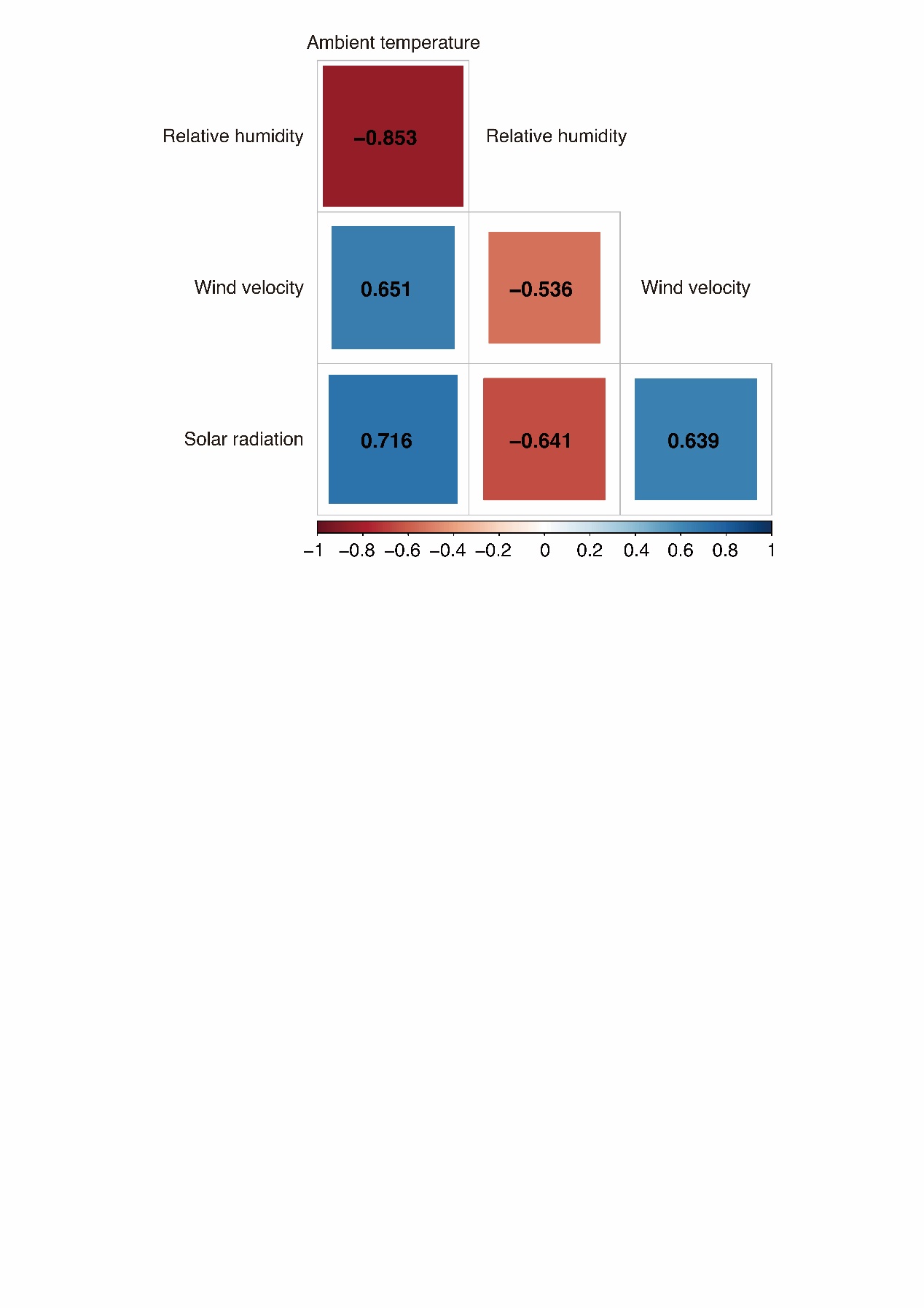


Fig. S2. Pearson correlation between all the abiotic variables (ambient temperature, relative humidity, wind velocity and solar radiation). Significant effects of correlation at P < 0.05 are presented in bold.

**
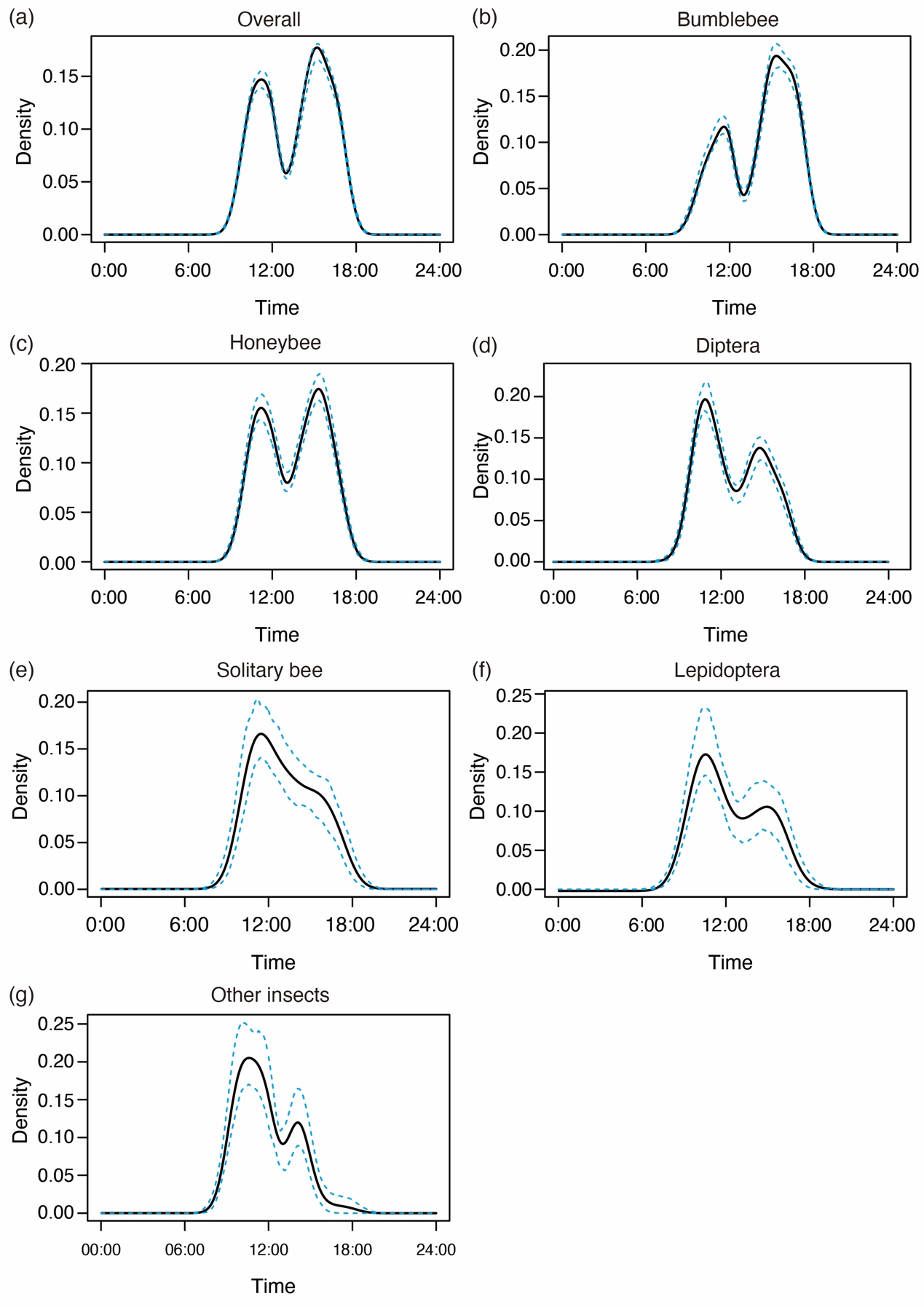
**

Fig. S3. Diurnal activity patterns in overall flower visitors (a), bumblebee (b), honeybee (c), Diptera (d), solitary bee (e), Lepidoptera (f), and other insects (g) in meadows across two flowering seasons on Yulong Snow Mountain, SW China. The black curves are fitted circular kernel distributions in the meadow, the blue dash curves are trend confidence interval.
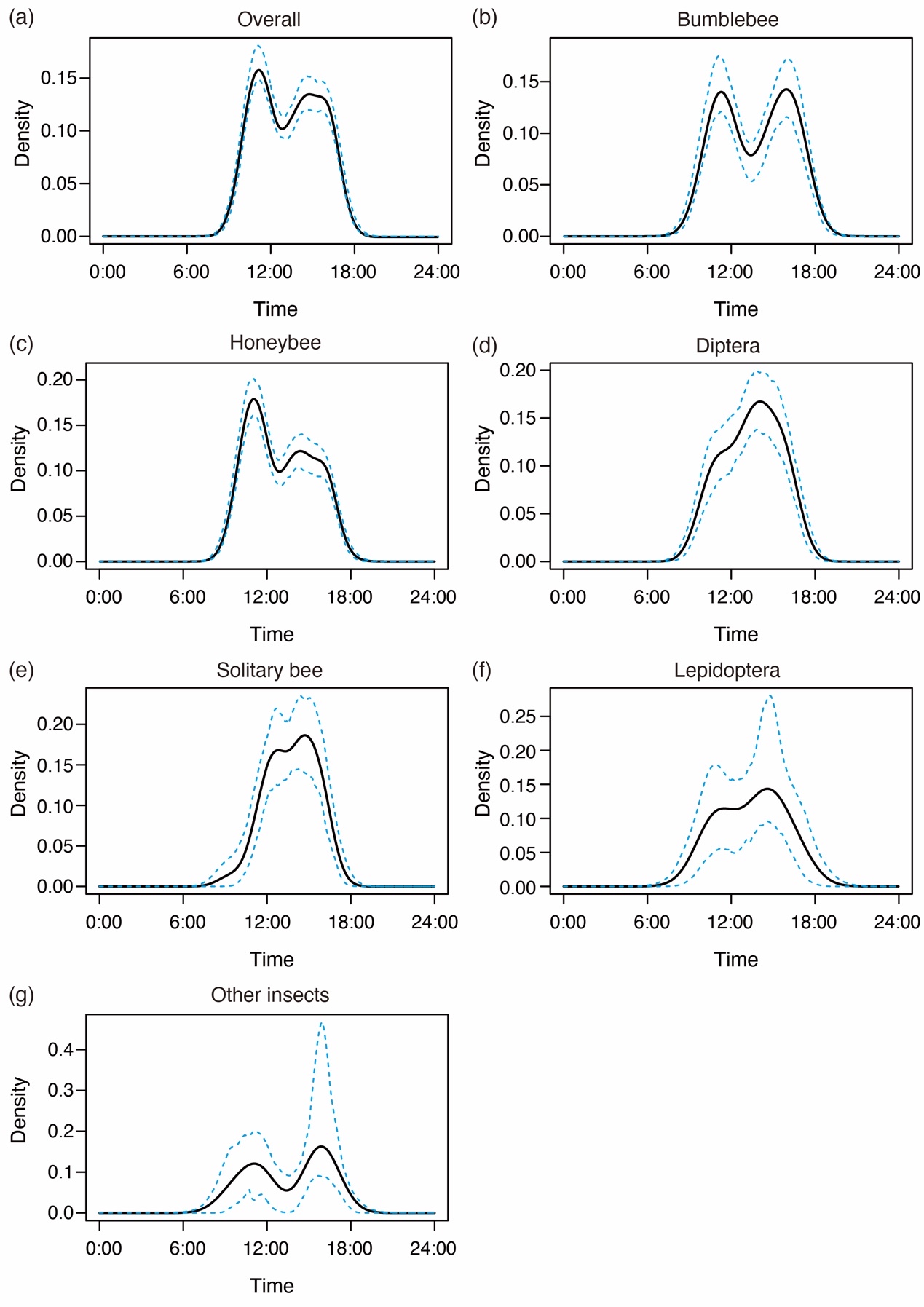


Fig. S4. Diurnal activity patterns in overall flower visitors (a), bumblebee (b), honeybee (c), Diptera (d), solitary bee (e), Lepidoptera (f), and other insects (g) in forests across two flowering seasons on Yulong Snow Mountain, SW China. The black curves are fitted circular kernel distributions in the forest, the blue dash curves are trend confidence interval.


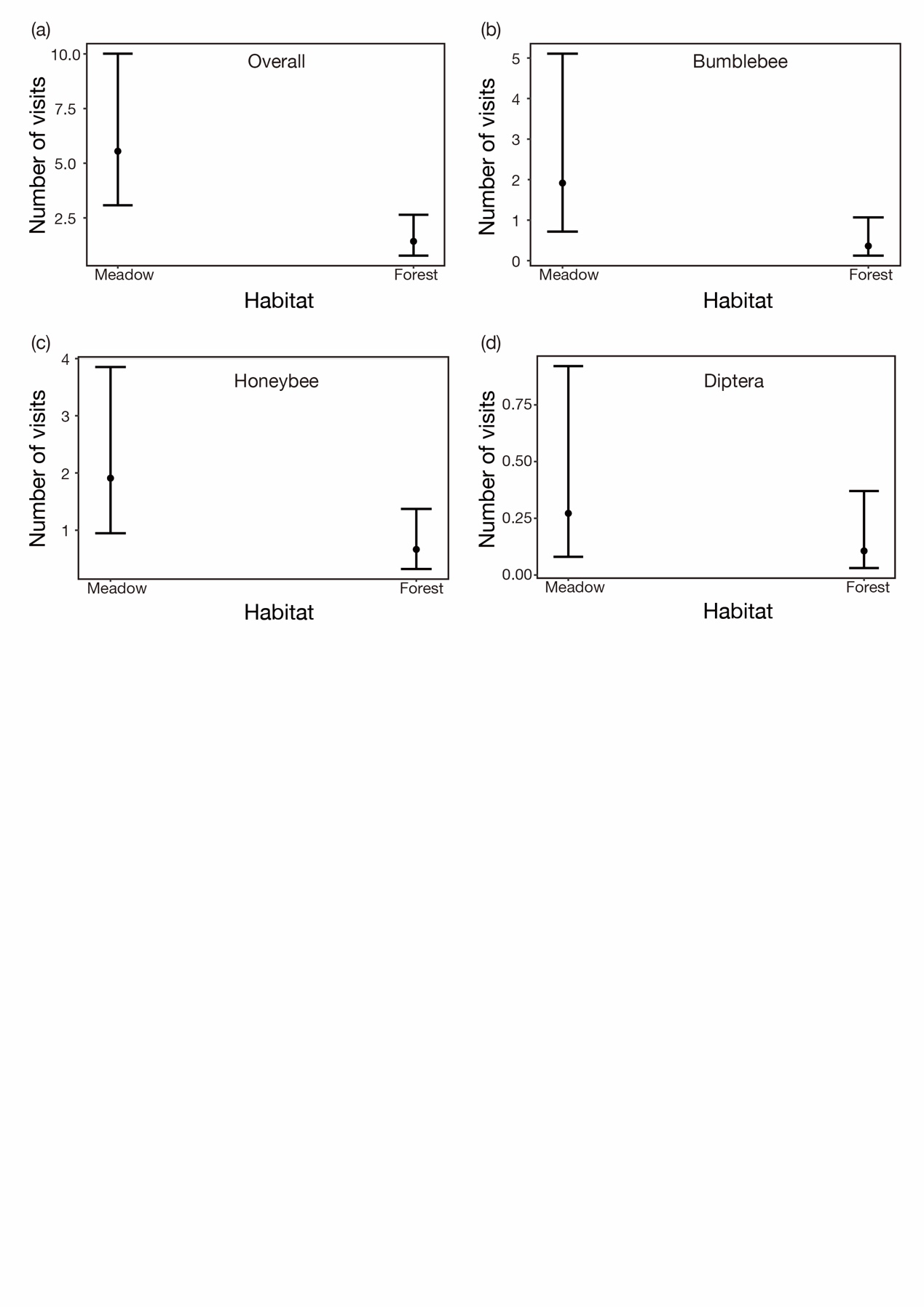


Fig. S5. The relationship between the number of visits and habitat types plotted from the results of the GLMMs model. Only functional groups with significant relationships are plotted. (a) overall flower visitors, (b) bumblebee, (c) honeybee and (d) Diptera.


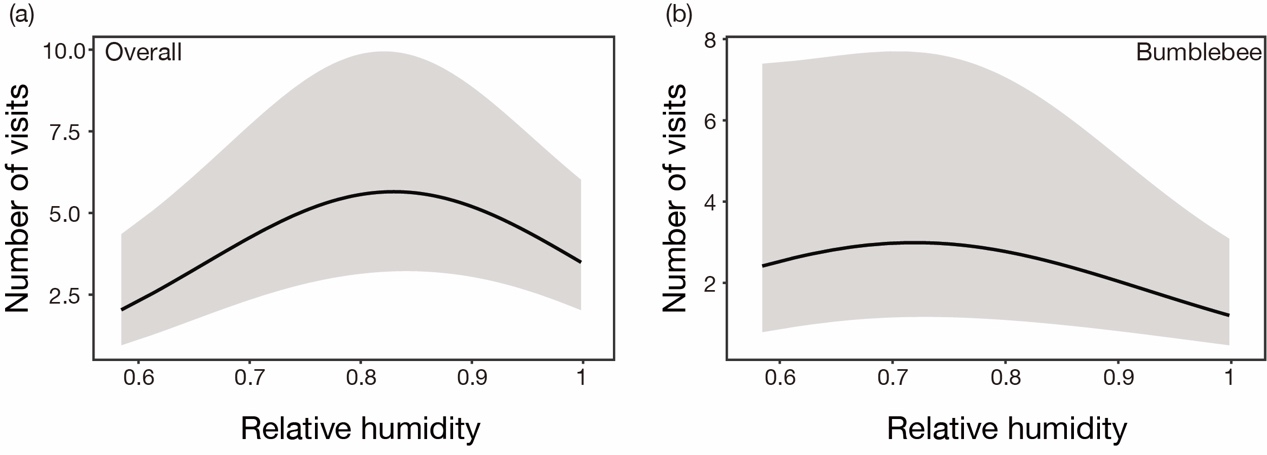


Fig. S6. The relationship between the number of visits and relative humidity plotted from the results of the GLMMs model. Only functional groups with significant relationships are plotted. (a) overall flower visitors, (b) bumblebee. The light grey area represents the 95% confidence interval.


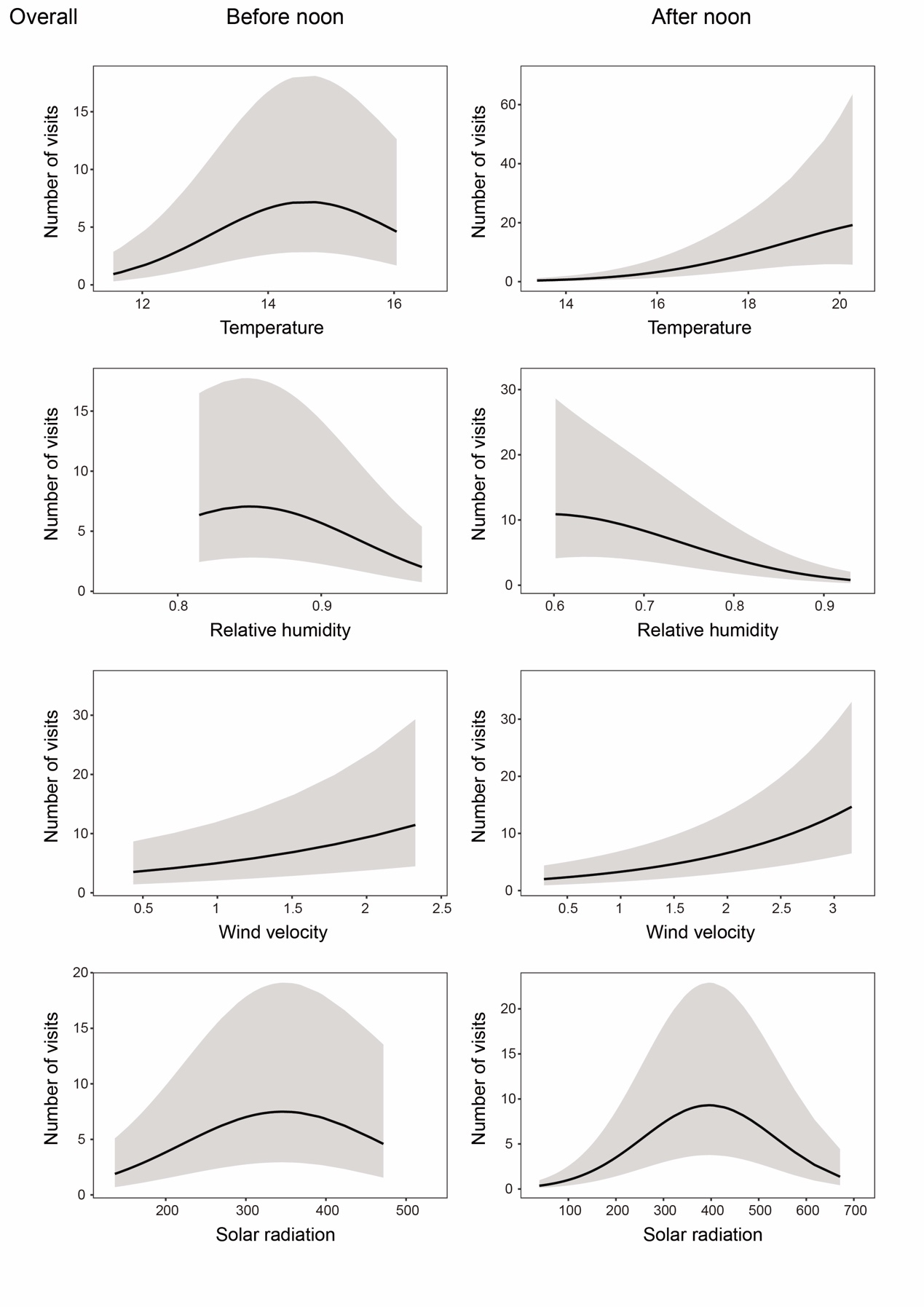


Fig. S7. The relationship between the number of overall visitor visits and environmental factors corresponding to the two periods plotted from the results of the GLMMs model. Only functional groups with significant relationships are plotted. The light grey area represents the 95% confidence interval.


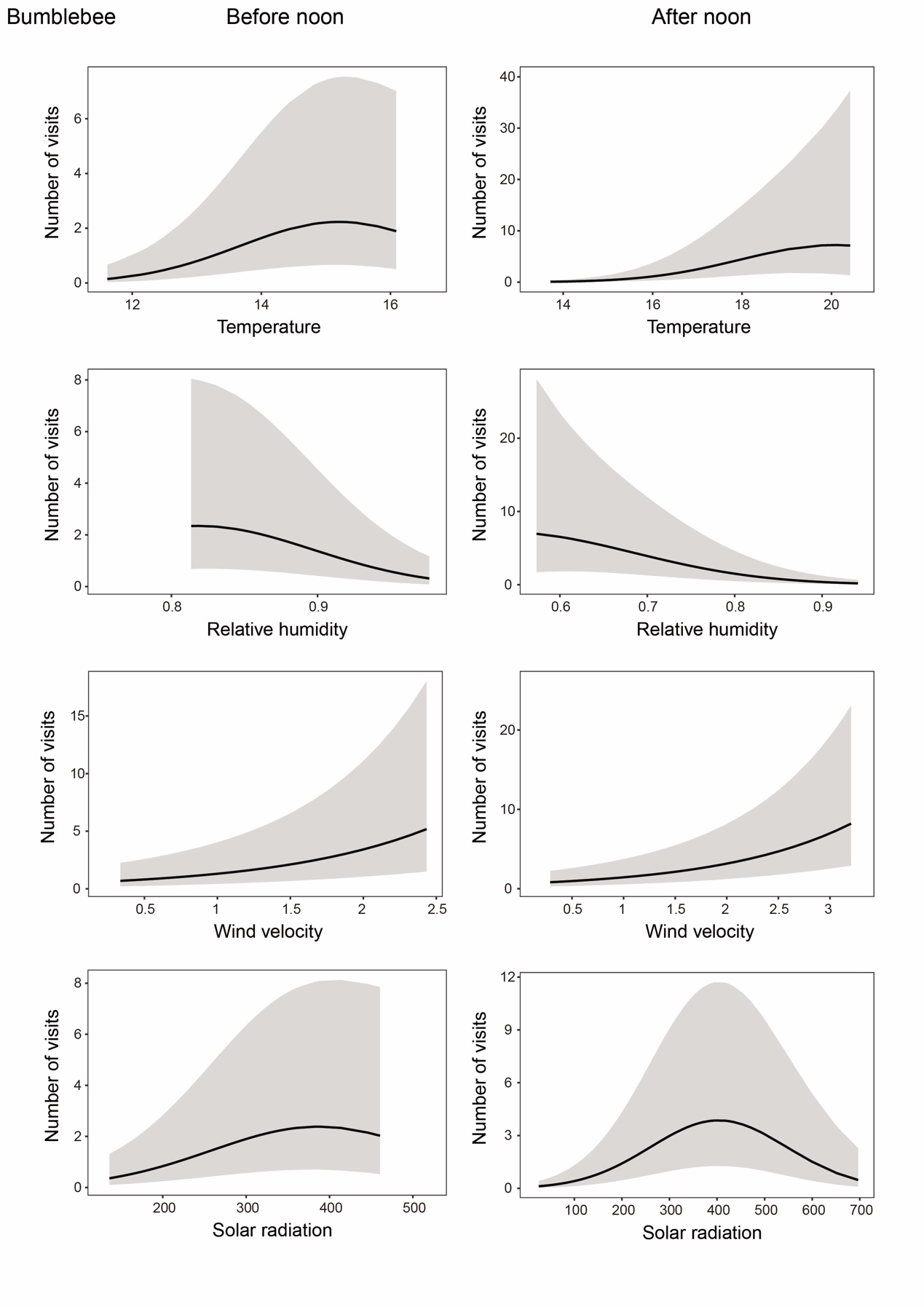


Fig. S8.The relationship between the number of bumblebee visits and environmental factors corresponding to the two periods plotted from the results of the GLMMs model. Only functional groups with significant relationships are plotted. The light grey area represents the 95% confidence interval.


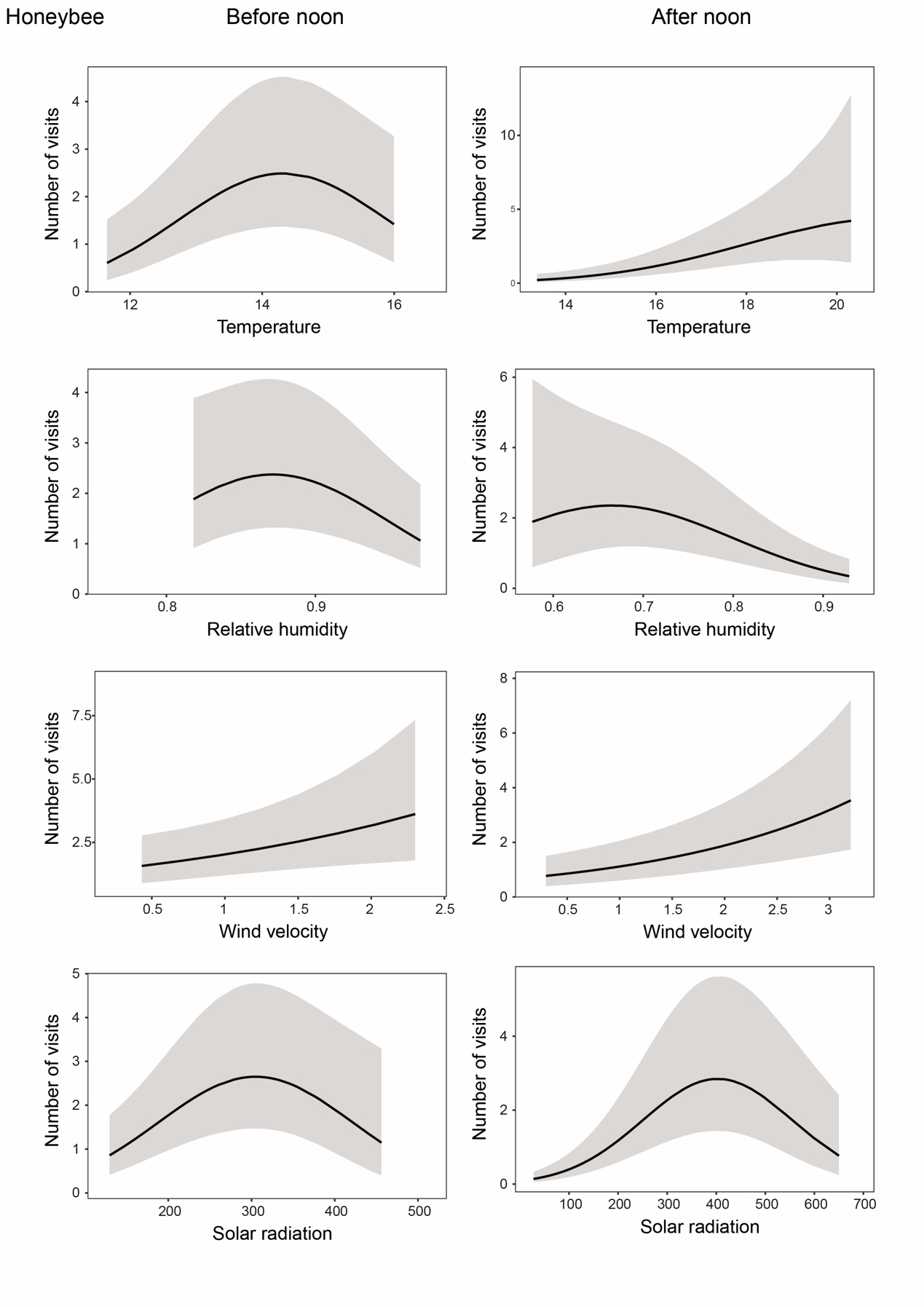


Fig. S9. The relationship between the number of honeybee visits and environmental factors corresponding to the two periods plotted from the results of the GLMMs model. Only functional groups with significant relationships are plotted. The light grey area represents the 95% confidence interval.


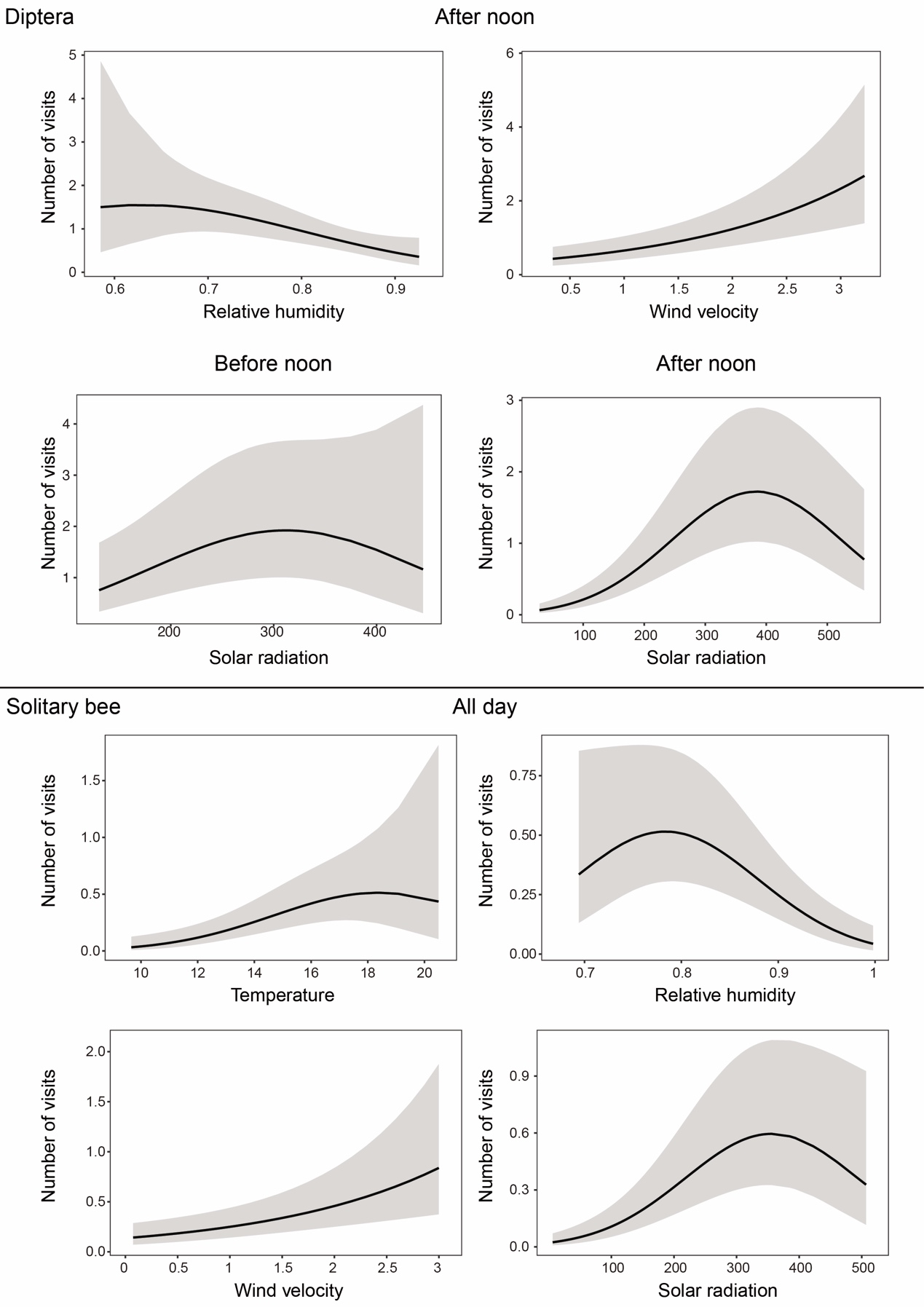


Fig. S10. The relationship between the number of Diptera and solitary bee visits and environmental factors corresponding to the two periods plotted from the results of the GLMMs model. Only functional groups with significant relationships are plotted. The light grey area represents the 95% confidence interval.
